# Influenza Vaccination Coverage and Determinants of New Vaccinations During the COVID-19 Pandemic in Spain (ENE-COVID): Nationwide Population-Based Study

**DOI:** 10.2196/60658

**Published:** 2025-07-01

**Authors:** Miguel Ángel de la Cámara, Nerea Fernández de Larrea-Baz, Roberto Pastor-Barriuso, Amparo Larrauri, Pablo Fernández-Navarro, Marina Pollán, Beatriz Pérez-Gómez, ENE-COVID Study Group

**Affiliations:** 1 National Centre for Epidemiology, Instituto de Salud Carlos III, Monforte de Lemos, 5, Madrid, Spain, 34 918222862; 2 Department of Educational Sciences, University of Alcalá, Alcalá de Henares, Spain; 3 Consortium for Biomedical Research in Epidemiology and Public Health (CIBERESP), Madrid, Spain

**Keywords:** influenza prevention, influenza vaccination, vaccination coverage, vaccination determinants, COVID-19 pandemic, influenza, vaccination, influenza vaccine, Spain, population-based study, health information, adult, ENE-COVID, epidemiology, sociodemographic, infection prevention, public health

## Abstract

**Background:**

Influenza vaccination coverage is commonly suboptimal. However, the COVID-19 pandemic and consequent high exposure to health information may have changed population attitudes toward this vaccination.

**Objective:**

The aim of this study is to describe influenza vaccine uptake in Spain during the first influenza season following the start of the COVID-19 pandemic compared to the previous one and identify characteristics associated with vaccination among those previously unvaccinated.

**Methods:**

This was a population-based study of 28,987 adults included in influenza vaccination target groups (≥65 years old, with risk conditions, living with someone with risk conditions, health care workers, security or emergency workers) who were participants in the nationwide Seroepidemiological Survey of SARS-CoV-2 Infection in Spain (ENE-COVID) study. Information on vaccination and sociodemographic, health, and COVID-19–related factors was collected by interview. Coverage change from 2019 to 2020 and standardized prevalences of vaccination in 2020 among the population unvaccinated in 2019 were estimated using logistic model–based methods.

**Results:**

Coverage rose from 31.4% (95% CI 30.5%‐32.2%) to 46.8% (95% CI 45.8%‐47.8%). People ≥65 years old showed the highest uptake in both periods (58.3%, 95% CI 56.8%‐59.8% and 74.8%, 95% CI 73.5%‐76.1%), while health care workers had the greatest increase (22%, 95% CI 17.8%‐26.2%). Among people unvaccinated in 2019, factors associated with vaccination in 2020 were age, female sex, higher education, Spanish nationality, multimorbidity, being a former smoker, obesity, contact with COVID-19 cases, living with older adults, living in provinces with low COVID-19 incidence, wearing a face mask during family meetings, and using surgical/FFP2 masks.

**Conclusions:**

This study provides nationwide representative estimates of influenza vaccination coverage, which clearly increased between 2019 and 2020 in the 5 target groups. However, coverage goals were attained only in the ≥65 year old group, highlighting the importance of reinforcing influenza vaccination. Our detailed results on determinants of vaccination provide some clues to tailor vaccination strategies.

## Introduction

Seasonal influenza is a global health problem, with almost one billion cases every year and 3‐5 million severe cases, causing 290,000‐650,000 deaths worldwide [1]. Influenza vaccination is the leading safe and effective pharmacological intervention available to prevent and reduce the impact of this annual pandemic, particularly in more susceptible groups [2], such as older people, pregnant women, and people with underlying comorbidities, as well as those in selected occupations, including health care workers (HCWs). However, in most countries, the established goal of 70%‐75% vaccination coverage for these target groups is not achieved [3-5].

Several factors may drive vaccine uptake. In addition to health policy and promotion strategies or socioeconomic factors (eg, household income or town size) [6], some individual characteristics seem to be associated to influenza vaccination [7,8]. Higher coverage has been reported in men, older people, people with higher education, those who make frequent visits to the doctor, or those with risk factors for severe influenza. Risk perception of influenza disease and an individual’s opinion about the efficacy and safety of the vaccine usually play a role in the decision to be vaccinated [7]. However, in 2020, a new actor came into play: the deadly COVID-19 pandemic, caused by another respiratory virus. That year, the World Health Organization emphasized the need for influenza vaccination [9], concerned by the additional burden of cocirculation of both viruses for vulnerable populations and health systems. Its recommendations were adopted by different countries, with reinforced influenza vaccination campaigns during the 2020/2021 season. These strengthened campaigns, together with the fear of a higher severity of potential influenza and SARS-CoV-2 coinfections and the commitment of the population to help to avoid a new overload of the health care system, probably contributed to increased influenza vaccination acceptance, as has been observed in some studies [10-15]. However, most of these studies relied on convenience samples, mostly gathered through web-based questionnaires, or explored only a limited number of population characteristics.

In 2020, the Spanish Ministry of Health and the Institute of Health Carlos III, in collaboration with the health services of all Spanish regions, launched the Seroepidemiological Survey of SARS-CoV-2 Infection in Spain (ENE-COVID), a nationwide, representative, population-based seroepidemiological survey, with more than 60,000 participants, to quantify SARS-CoV-2 infection prevalence [16]. In the fourth round of the ENE-COVID, in November 2020, self-reported information on influenza vaccination was gathered, together with a wide range of sociodemographic, health, and COVID-19–related epidemiological data.

Taking advantage of this sound design, the aims of this study are to provide influenza vaccination coverage estimates among the adult population corresponding to the main target groups in Spain in 2019 and 2020 (ie, the influenza season prior to the start of the COVID-19 pandemic and the first influenza season following the start of the pandemic) and to identify a wide range of factors related to the change from not being vaccinated to influenza vaccine uptake.

## Methods

### Study Design and Population

ENE-COVID is a nationwide population-based cohort study designed to provide representative seroprevalence figures for SARS-CoV-2 infection in Spain for the noninstitutionalized population [16]. Briefly, 35,885 households were selected through a stratified 2-stage sampling, with strata formed by cross-classifying the 52 Spanish provinces and autonomous cities with 4 categories of municipality size. A total of 1500 census tracts were selected with probability proportional to their size, and then 24 households were randomly sampled within each census tract. Households were contacted by telephone and all residents were invited to participate. Participation required answering an epidemiological questionnaire and being assessed for SARS-CoV-2 antibodies by a point-of-care test. The survey was repeated on 4 occasions for the same cohort between April and November 2020 [17].

For this study, we analyzed data from the fourth follow-up round, conducted in November 16-29, 2020, which included 51,409 participants. Among them, adult participants in any of the following influenza vaccination target groups were included in the present analysis (28,987 participants): (1) people ≥65 years old; (2) people aged 18‐64 years reporting health conditions that increase their risk of severe influenza, including diabetes; chronic lung, liver, or kidney disease; asthma, cardiovascular disease, hematological tumour, any other cancer in the last five years, immunosuppression treatment, or severe obesity; (3) people living with someone reporting any of these health conditions; (4) HCWs, including health care or sociosanitary workers, and at-home professional caregivers; and (5) security and emergency workers. For participants that pertained to more than one group, the following order of preference was applied: age ≥65 years, risk conditions, HCW, living with someone with a risk condition, and security and emergency workers. Other population groups included in the international influenza vaccination recommendations, such as pregnant women or children with certain conditions, were not addressed because the questionnaire information did not allow us to comprehensively identify participants in those groups.

### Epidemiological Information

The epidemiological questionnaire included information on sociodemographic characteristics (registered sex, age, nationality, education, employment status, and job sector), health-related characteristics (self-reported height, weight, smoking habit, health status, chronic conditions, and officially recognized disability, that is, having received a certificate of any degree of disability—physical, sensory, intellectual, or mental disability—after evaluation by the technical teams of the corresponding regional authority), and COVID-19–related factors (self-reported COVID-19 pneumonia or hospitalization, contacts with symptomatic or confirmed cases, and behaviors related to possible exposure to SARS-CoV-2 such as mask wearing or attending family events). We classified personal experience of COVID-19 as no infection, not severe infection (positive antibody test or self-reported positive polymerase chain reaction or antigen test, without pneumonia or hospitalization), or severe infection (pneumonia or hospitalization). Additionally, we considered household and contextual information: household size and age composition (presence of children under 3 years of age or adults over 65 years of age), autonomous community (administrative level at which regional influenza vaccination campaigns are organized), municipality size, average personal income in the census tract relative to each province, and provincial 7-day cumulative incidence of COVID-19 at the start of the field work (November 16-22, 2020).

Self-reported influenza vaccination was collected by asking the participants if they had been vaccinated in the fall of 2020 or intended to do so and if they had received the vaccine in the previous year. Since the 2020‐2021 vaccination campaign was still ongoing at the time of interview, participants who reported that they intended to be vaccinated were considered as vaccinated in our main analyses (a sensitivity analysis excluding these participants was also performed).

### Statistical Analysis

Influenza vaccination coverage in 2019 and 2020 was calculated as the proportion of participants who reported having been vaccinated or intended to do so in that year. Vaccination coverage by sociodemographic, health, and COVID-19–related characteristics was standardized to the overall distribution of influenza vaccination target groups in the entire study population and compared by estimating standardized differences between 2019 and 2020. We also computed target group–standardized prevalences of influenza vaccination by cross-classifying 2019 and 2020 data (not vaccinated in any season, vaccinated only in 2019, vaccinated both years, or only vaccinated in 2020) overall and by participant characteristics. Logistic model-based standardization methods were used for this purpose [18].

To assess factors associated with a change from 2019 to 2020 favouring vaccination, we calculated the prevalence of new influenza vaccination in 2020 among participants unvaccinated in 2019. Using logistic model–based standardization methods [18], the prevalence of new vaccinees by participant characteristics was standardized to the overall distribution of influenza vaccination groups, sex, age, education, and autonomous community in the unvaccinated target population in 2019. Standardized prevalence differences and ratios for new influenza vaccination were estimated across categories of participant characteristics. Lastly, to assess potential effect modifications of prespecified characteristics on new vaccination by target group, we included target group-by-covariate interactions in logistic models and estimated standardized prevalence ratios (SPR) within each target group. We tested for homogeneity using Wald tests.

All statistical analyses used sampling weights to correct for the different selection probabilities by province and the distinct response rates by sex, age, and census tract income, and they accounted for stratification and clustering by household and census tract when computing CIs and tests [16]. Analyses were performed in Stata (version 16; StataCorp) and graphics were produced in R (version 4; R Foundation for Statistical Computing).

### Ethical Considerations

The institutional review board of the Institute of Health Carlos III approved the study (register number PI 39_2020) and written informed consent was obtained from all study participants. Data were deidentified before statistical analysis. No compensation was provided to participants.

## Results

**Figure 1.**
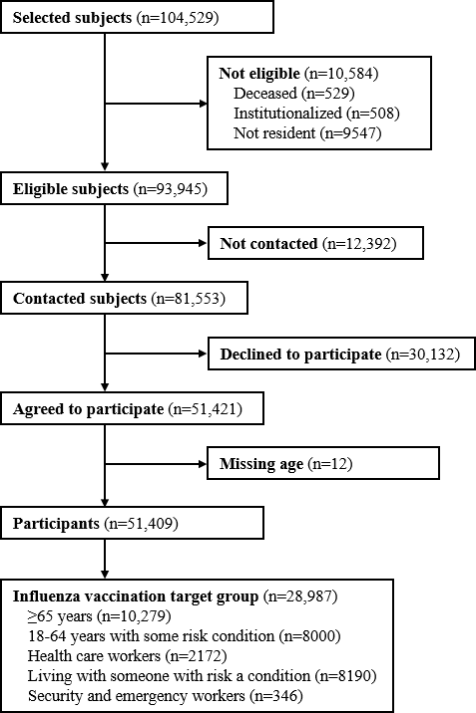
Flowchart of participants in the fourth round of the ENE-COVID study, November 16‐29, 2020, Spain. ENE-COVID: Seroepidemiological Survey of SARS-CoV-2 Infection in Spain.

Figure 1 summarizes the participation flowchart for the fourth round of ENE-COVID. The 28,987 adults included in the study population were distributed by vaccination target groups: aged ≥65 years (10,279/28,987, 35.5%), living with someone with risk conditions (8190/28,987, 28.2%), aged <65 years with a risk condition (8000/28,987, 27.6%), HCW (2172/28,987, 7.5%), and security and emergency workers (346/28,987, 1.2%). Among them, 15,802 (54.5%) were women (mean age 56.7, SD 17.6 years) and 13,185 (45.5%) were men (mean age 56.3, SD 17.4 years).

Table 1 summarizes influenza vaccination coverage in 2019 and 2020 by target group and participants’ characteristics. Overall coverage in 2019 was 31.4% (95% CI 30.5%‐32.2%) and it was 46.8% (45.8%‐47.8%) in 2020, corresponding to an absolute increase of 15.4% (14.1%‐16.8%) and a relative increase of 49% (44%-54%). By target group, the highest uptake was among adults ≥65 years old, with 58.3% (56.8%‐59.8%) in 2019, achieving 74.8% (73.5%‐76.1%) in 2020, while the lowest figures were observed among people living with someone with risk conditions (2019: 9.3%, 8.4%‐10.1%; 2020: 21.1%, 19.7%‐22.4%) and security/emergency workers (2019: 8.6%, 4.7%‐12.4%; 2020: 21%, 15.2%‐26.8%). All groups had a marked increase in 2020, especially HCWs (22.0%, 17.8%‐26.2%).

**Table 1.** Influenza vaccination coverage in 2019 and 2020^a^ and coverage differences among adults of selected vaccination target groups^b^ (Spain, November 2020).

	Number	2019 coverage, % (95% CI)^c^	2020 coverage, % (95% CI)^c^	Standardized difference, % (95% CI)^c^
Total	28,987	31.4 (30.5‐32.2)	46.8 (45.8‐47.8)	15.4 (14.1‐16.8)
**Influenza-related characteristics**				
* **Influenza vaccination target group***				
≥65 years	10,279	58.3 (56.8‐59.8)	74.8 (73.5‐76.1)	16.5 (14.5‐18.5)
<65 years with a risk condition^d^	8000	25.0 (23.7‐26.3)	41.4 (39.8‐43.0)	16.4 (14.3‐18.4)
Health care workers	2172	23.3 (20.6‐26.0)	45.3 (42.2‐48.5)	22.0 (17.8‐26.2)
Living with someone with a risk condition^d^	8190	9.3 (8.4‐10.1)	21.1 (19.7‐22.4)	11.8 (10.2‐13.4)
Security and emergency workers	346	8.6 (4.7‐12.4)	21.0 (15.2‐26.8)	12.4 (5.5‐19.4)
* **Number of*** ***influenza vaccination criteria***				
1	16,583	28.0 (26.8‐29.2)	44.3 (43.0‐45.5)	16.3 (14.5‐18.0)
2	9321	32.7 (31.3‐34.0)	48.5 (46.9‐50.2)	15.9 (13.8‐18.0)
3	3083	40.9 (38.4‐43.3)	56.6 (53.4‐59.8)	15.7 (11.7‐19.8)
**Sociodemographic characteristics**				
* **Sex***				
Men	13,185	30.9 (29.9‐31.9)	45.2 (43.9‐46.4)	14.3 (12.7‐15.9)
Women	15,802	31.8 (30.7‐32.8)	48.2 (47.0‐49.4)	16.4 (14.9‐18.0)
* **Age group*** ***(years)***				
18‐34	4059	10.7 (9.4‐12.0)	20.2 (18.5‐22.0)	9.5 (7.3‐11.8)
35‐59	11,920	17.1 (16.1‐18.0)	33.0 (31.7‐34.3)	15.9 (14.3‐17.5)
60‐64	2729	34.7 (32.1‐37.2)	57.1 (54.5‐59.8)	22.5 (18.8‐26.2)
65‐69	3339	46.8 (44.3‐49.3)	70.8 (68.8‐72.9)	24.1 (20.8‐27.3)
70‐74	2799	56.1 (53.4‐58.8)	74.3 (71.8‐76.8)	18.2 (14.6‐21.9)
75‐79	1988	64.9 (62.1‐67.7)	75.8 (73.3‐78.4)	11.0 (7.1‐14.8)
≥80	2153	74.0 (71.3‐76.8)	81.0 (78.6‐83.3)	6.9 (3.3‐10.5)
* **Nationality***				
Spanish	28,172	31.6 (30.7‐32.4)	47.2 (46.1‐48.2)	15.6 (14.2‐16.9)
Other	815	24.0 (19.9‐28.1)	35.3 (31.1‐39.5)	11.3 (5.4‐17.2)
* **Education***				
Primary or less	7830	35.2 (33.7‐36.6)	49.0 (47.3‐50.7)	13.8 (11.6‐16.1)
High school/vocational education	15,191	29.0 (27.9‐30.1)	44.5 (43.3‐45.8)	15.5 (13.8‐17.2)
University	5745	31.9 (30.0‐33.7)	49.9 (48.1‐51.7)	18.0 (15.4‐20.6)
* **Employment status***				
Actively working	11,730	23.8 (22.2‐25.4)	40.7 (38.9‐42.4)	16.9 (14.5‐19.3)
Student	974	19.0 (14.3‐23.6)	29.8 (25.0‐34.6)	10.8 (4.1‐17.5)
Retired	10,636	39.1 (37.4‐40.8)	57.3 (55.3‐59.3)	18.2 (15.6‐20.8)
Unemployed/other	5647	29.5 (27.8‐31.1)	44.6 (42.7‐46.5)	15.1 (12.6‐17.7)
* **On-site activity***				
No	16,267	34.7 (33.6‐35.9)	50.6 (49.3‐51.9)	15.8 (14.1‐17.6)
On-site work	10,847	25.8 (24.2‐27.3)	43.4 (41.8‐45.0)	17.6 (15.4‐19.8)
Other on-site activity	1873	25.0 (21.9‐28.1)	36.3 (33.0‐39.5)	11.3 (6.8‐15.8)
**Household and residence characteristics**				
* **Living with older*** ***adults***				
No	18,778	29.6 (28.6‐30.6)	44.6 (43.4‐45.7)	15.0 (13.5‐16.4)
Yes	10,195	34.3 (33.0‐35.7)	51.5 (49.8‐53.2)	17.2 (15.0‐19.4)
* **Living with*** ***children*** ***<3*** ***years***				
No	27,792	31.3 (30.4‐32.1)	46.8 (45.7‐47.8)	15.5 (14.1‐16.9)
Yes	1195	33.6 (29.4‐37.7)	46.8 (42.9‐50.7)	13.2 (7.6‐18.9)
* **Household size***				
1‐2 people	11,816	32.2 (31.0‐33.4)	48.5 (47.0‐49.9)	16.3 (14.4‐18.1)
≥3 people	17,171	30.6 (29.5‐31.8)	45.7 (44.4‐46.9)	15.0 (13.3‐16.8)
* **Municipality size*** ***(population)***				
>100,000	8727	32.0 (30.5‐33.4)	47.6 (45.9‐49.3)	15.7 (13.5‐17.8)
20,000‐100,000	8495	30.5 (29.1‐31.9)	46.1 (44.3‐47.9)	15.5 (13.3‐17.8)
5000‐20,000	6075	30.2 (28.4‐31.9)	46.0 (44.2‐47.8)	15.9 (13.4‐18.3)
<5000	5690	33.0 (31.4‐34.5)	46.8 (44.3‐49.4)	13.9 (10.9‐16.9)
* **Relative income of the residential area***				
Under 25th percentile	7757	30.6 (29.1‐32.1)	46.2 (44.1‐48.2)	15.6 (13.1‐18.1)
25th to 50th percentile	7272	33.1 (31.4‐34.7)	47.2 (45.3‐49.1)	14.2 (11.6‐16.7)
50th to 75th percentile	6517	30.0 (28.6‐31.4)	44.7 (42.8‐46.5)	14.7 (12.3‐17.0)
Over 75th percentile	7441	31.7 (30.0‐33.5)	49.1 (47.1‐51.1)	17.3 (14.7‐20.0)
**Health-related characteristics**				
* **Self-rated health***				
Very good or good	24,451	29.9 (29.0‐30.8)	45.7 (44.6‐46.8)	15.8 (14.4‐17.2)
Bad or very bad	4536	38.4 (36.7‐40.1)	52.9 (51.0‐54.9)	14.6 (12.0‐17.1)
* **Chronic diseases***				
None	10,720	22.5 (21.1‐23.9)	38.1 (36.5‐39.8)	15.6 (13.5‐17.8)
1	7646	29.9 (28.6‐31.3)	47.1 (45.5‐48.6)	17.1 (15.1‐19.2)
2‐3	8451	37.2 (35.7‐38.7)	53.9 (52.1‐55.7)	16.6 (14.3‐19.0)
≥4	2170	49.9 (47.1‐52.6)	63.9 (61.0‐66.8)	14.1 (10.1‐18.1)
* **Disability***				
No	26,688	30.7 (29.8‐31.6)	46.1 (45.1‐47.2)	15.4 (14.0‐16.8)
Yes	2118	39.8 (37.2‐42.4)	55.7 (52.8‐58.7)	16.0 (12.1‐19.9)
* **BMI***				
Underweight and normal weight	10,986	29.6 (28.5‐30.8)	45.0 (43.6‐46.4)	15.3 (13.5‐17.2)
Overweight	11,425	31.7 (30.6‐32.9)	47.2 (45.8‐48.6)	15.5 (13.6‐17.3)
Obesity	6571	33.5 (32.0‐35.0)	49.4 (47.7‐51.1)	15.9 (13.6‐18.1)
* **Tobacco use***				
Never	14,848	32.0 (30.9‐33.0)	46.7 (45.4‐48.0)	14.8 (13.1‐16.4)
Former	8073	34.3 (33.0‐35.7)	52.5 (50.9‐54.0)	18.1 (16.1‐20.2)
Current	6064	25.0 (23.5‐26.5)	39.9 (38.1‐41.7)	14.9 (12.5‐17.2)
**COVID-19–related characteristics**				
* **Personal history***				
No infection	24,800	31.5 (30.6‐32.4)	47.0 (46.0‐48.1)	15.5 (14.1‐16.9)
Not-severe infection	3445	30.3 (28.2‐32.4)	44.7 (42.0‐47.3)	14.4 (11.0‐17.7)
Pneumonia or hospitalization	742	32.0 (28.2‐35.7)	49.1 (44.2‐53.9)	17.1 (10.9‐23.2)
* **Contact with case***				
No contact	18,584	31.9 (30.9‐32.9)	46.6 (45.4‐47.8)	14.7 (13.1‐16.2)
Noncohabitant symptomatic contact	1254	26.4 (23.2‐29.6)	44.7 (41.1‐48.2)	18.3 (13.5‐23.1)
Noncohabitant case contact	4325	30.6 (28.6‐32.5)	47.7 (45.8‐49.6)	17.1 (14.4‐19.9)
Household symptomatic contact	1380	29.9 (26.3‐33.5)	46.4 (42.8‐50.0)	16.5 (11.4‐21.5)
Household case contact	3444	31.9 (29.6‐34.2)	47.8 (45.0‐50.6)	15.9 (12.3‐19.6)
** *****Cumulative*** ***provincial*** ***7-day*** ***incidence (per 100,000)***				
<100	2331	27.5 (25.3‐29.7)	41.9 (39.1‐44.7)	14.4 (10.8‐17.9)
100‐200	18,378	32.0 (31.0‐33.0)	47.3 (46.1‐48.5)	15.3 (13.7‐16.9)
200‐300	7447	30.2 (28.5‐31.9)	46.5 (44.3‐48.7)	16.3 (13.5‐19.0)
≥300	831	35.1 (31.2‐39.0)	49.9 (45.4‐54.3)	14.8 (8.9‐20.7)
***Number of people in close contact***				
None	3020	33.7 (31.3‐36.1)	47.1 (44.3‐49.9)	13.4 (9.7‐17.1)
1‐2	8788	32.3 (31.0‐33.6)	48.7 (47.1‐50.3)	16.4 (14.3‐18.5)
3‐5	11,372	31.4 (30.2‐32.7)	47.0 (45.6‐48.3)	15.5 (13.7‐17.4)
6‐9	4328	29.0 (27.1‐30.8)	45.5 (43.5‐47.5)	16.5 (13.7‐19.2)
≥10	1479	26.9 (23.5‐30.2)	39.4 (36.1‐42.7)	12.5 (7.9‐17.2)
***Face*** ***mask type used***				
Cloth	4312	28.4 (26.6‐30.2)	40.5 (38.6‐42.4)	12.1 (9.5‐14.7)
Surgical	18,449	30.8 (29.8‐31.8)	46.4 (45.2‐47.6)	15.6 (14.1‐17.2)
FFP2	6170	34.7 (33.1‐36.2)	52.2 (50.4‐54.0)	17.5 (15.1‐19.9)
***Face*** ***mask use in family meetings***				
No	9850	29.1 (27.9‐30.4)	43.0 (41.6‐44.4)	13.8 (11.9‐15.7)
Sometimes	2618	30.3 (28.1‐32.5)	46.1 (43.6‐48.7)	15.9 (12.5‐19.2)
Yes	11,852	33.2 (31.9‐34.4)	49.3 (47.8‐50.7)	16.1 (14.2‐18.0)
Did not meet	4667	32.4 (30.7‐34.1)	49.9 (47.8‐52.1)	17.5 (14.8‐20.2)
***Indoor bar attendance***				
<Once/month	21,629	32.5 (31.5‐33.4)	47.9 (46.7‐49.0)	15.4 (13.9‐16.9)
1‐3 times/month	3938	27.6 (25.7‐29.5)	44.0 (41.9‐46.0)	16.4 (13.6‐19.2)
≥Once/week	3420	28.3 (26.1‐30.4)	43.5 (41.2‐45.9)	15.3 (12.1‐18.5)
***Indoor family meetings with >10*** ***people***				
Never	25,078	31.7 (30.8‐32.6)	47.3 (46.2‐48.4)	15.6 (14.2‐17.0)
1‐5 times	3098	28.7 (26.4‐31.1)	43.3 (40.9‐45.8)	14.6 (11.2‐18.0)
>5 times	811	30.7 (26.3‐35.1)	44.4 (40.1‐48.7)	13.7 (7.6‐19.8)
***Indoor events, most people without mask***				
No	26,482	31.6 (30.7‐32.4)	47.2 (46.1‐48.3)	15.6 (14.2‐17.0)
Yes	2505	29.3 (26.8‐31.8)	42.9 (40.3‐45.5)	13.6 (10.1‐17.2)
***Use of public transport***				
No	24,045	31.8 (30.9‐32.7)	47.4 (46.3‐48.5)	15.7 (14.2‐17.1)
Yes	4942	29.8 (28.1‐31.5)	44.3 (42.4‐46.3)	14.6 (12.0‐17.2)

a The 2020 campaign was not finished when the survey took place; in this table, participants answering that they had still not received the vaccine but intended to be vaccinated were considered vaccinated.

b Selected target groups: population older than 65 years, health care or sociosanitary workers or at-home caregivers, population with some risk condition, living with someone with some risk condition, and security and emergency workers.

c Standardized to the overall distribution of the influenza vaccination target group in the whole study population, except coverage for age groups that were not standardized.

d Risk conditions: diabetes; chronic lung, liver, or kidney disease; asthma, cardiovascular disease, hematological neoplasm, any other cancer in the last 5 years, immunosuppressor treatment, or severe obesity.

According to sociodemographic characteristics, in both influenza seasons, the standardized prevalence of vaccine uptake was higher among those with lower or higher educational level (compared to intermediate level), Spanish nationality, retired, or those living with older people, without clear differences by municipality size. On the other hand, in 2020, coverage was higher among women, and also somewhat higher in those residing in wealthier areas. Focusing on health-related factors, in both seasons, coverage was higher in those with bad or very bad self-rated health, disability, or obesity, and increased with the number of chronic diseases. Regarding tobacco use, the highest prevalence of vaccinees and the highest absolute increase in coverage (18.1%, 95% CI 16.1%‐20.2%) corresponded to former smokers. For COVID-19–related factors, people reporting pneumonia, contact with noncohabitant suspected or confirmed cases, using an FFP2 face mask, or not attending family meetings showed a higher absolute increase in coverage, while no differences were observed by provincial COVID-19 incidence.

**Figure 2.**
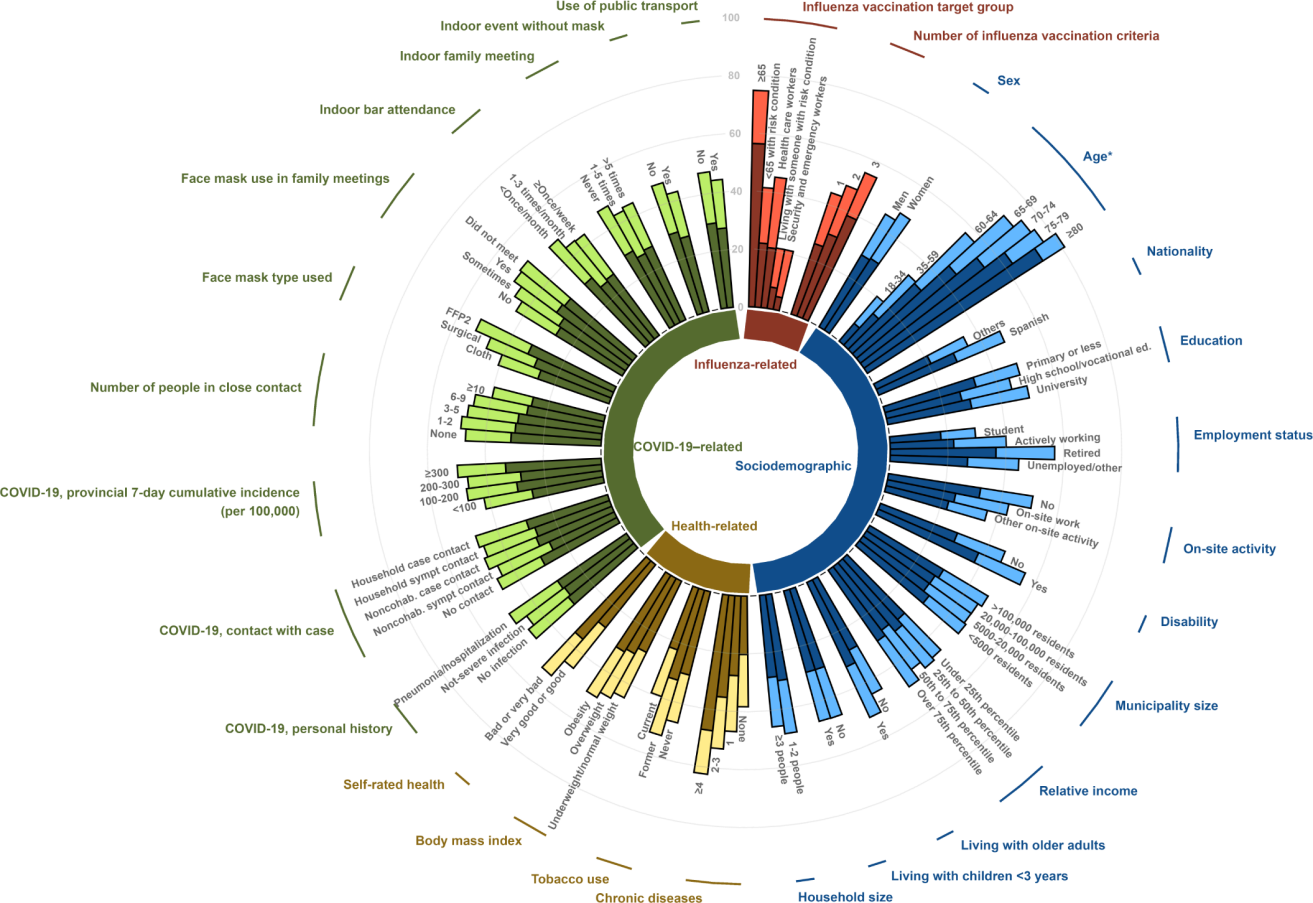
Prevalences of influenza vaccination, standardized by target group distribution in the overall population, by influenza-related, sociodemographic, health-related, and COVID-19–related factors. Dark colors represent the proportion of participants vaccinated in both the 2019 and 2020 campaigns, and light colors represent those vaccinated only in 2020 (new vaccinees). Noncohab: noncohabitant. *Prevalence by age not standardized.

Figure 2 shows a general picture of vaccination coverage in 2020, highlighting the contribution of the new vaccinees (those not receiving the vaccine in 2019), by participant characteristics, standardized to the overall vaccination target group composition. In all, 17.6% (95% CI 16.9%‐18.3%) of the participants were new vaccinees, ranging from 8.9% (7.1%‐10.7%) in the oldest age group to around 25% for those aged 60‐69 years and HCWs (Table S1 in Multimedia Appendix 1).

One of 4 participants unvaccinated in 2019 opted for influenza vaccination in 2020 (Table 2). This change was most prevalent, once standardized by sex, education, and autonomous community, among participants older than 65 years (44.8%, 95% CI 42.2%‐47.3%), followed by HCWs (30.0%, 95% CI 26.7%‐33.2%). On the other hand, among those living with someone with risk conditions, only 15% of unvaccinated individuals changed their mind (95% CI 13.8%‐16.2%). Regarding other factors, our results, additionally standardized by age and influenza vaccination target group, showed that this behavior change was more frequent in participants meeting more than one influenza vaccination criteria (SPR_two vs one_: 1.10, 95% CI 1.01‐1.19), women (SPR: 1.15, 95% CI 1.08‐1.22), older participants (SPR_65-69 vs 18-34_: 3.66, 95% CI 3.20‐4.19), those with higher education (SPR_university vs primary_: 1.28, 95% CI 1.15‐1.42), those with chronic diseases (SPR_≥4 vs none_: 1.35, 95% CI 1.15‐1.58), former smokers (SPR_former vs never_: 1.13, 95% CI 1.05‐1.22), or those living with older people (SPR: 1.19, 95% CI 1.11‐1.28). With respect to COVID-19–related factors, SARS-CoV-2 infection was not clearly associated with influenza vaccination, while being aware of previous contact with COVID-19 cases increased new vaccination (SPR_nonhousehold case contact vs no contact_: 1.14, 95% CI 1.04‐1.24). Curiously, local incidence of COVID-19 at interview was inversely related to the proportion of new vaccinees (SPR_highest vs lowest_: 0.79, 95% CI 0.59‐1.05). Regarding compliance with COVID-19 prevention measures, the use of masks in family meetings (SPR_always vs no_: 1.19, 95% CI 1.09‐1.30) and the type of mask worn (SPR_FFP2 vs cloth_: 1.43, 95% CI 1.28‐1.60) showed a remarkable association with vaccination.

**Table 2.** Prevalence of new influenza vaccination in 2020^a^ among participants unvaccinated in 2019, by sociodemographic, health-related, and COVID-19–related factors.

	Number	Crude prevalence, % (95% CI)	Standardized prevalence, % (95% CI)^b^	Standardized prevalence difference, % (95% CI)^b^	Standardized prevalence ratio, (95% CI)^b^
Total	19,334	25.7 (24.7 to 26.7)			
**Influenza-related characteristics**					
***Influenza vaccination target group***					
≥65 years	4214	44.1 (41.8 to 46.4)	44.8 (42.2 to 47.3)	Reference	Reference
<65 years with a risk condition	5880	25.7 (24.0 to 27.3)	25.7 (24.1 to 27.4)	–19.0 (–21.9 to –16.1)	0.58 (0.53 to 0.62)
Health care workers	1621	31.8 (28.6 to 35.1)	30.0 (26.7 to 33.2)	–14.8 (–18.9 to –10.7)	0.67 (0.59 to 0.76)
Living with someone with a risk condition	7309	15.0 (13.8 to 16.2)	14.9 (13.7 to 16.2)	–29.8 (–32.6 to –27.0)	0.33 (0.30 to 0.37)
Security and emergency workers	310	17.6 (11.8 to 23.3)	19.3 (13.3 to 25.4)	–25.4 (–32.0 to –18.9)	0.43 (0.31 to 0.59)
***Number of influenza vaccination criteria***					
1	12,992	21.3 (20.3 to 22.3)	24.7 (23.5 to 25.8)	Reference	Reference
2	5244	33.7 (31.6 to 35.7)	27.0 (25.2 to 28.9)	2.4 (0.2 to 4.5)	1.10 (1.01 to 1.19)
3	1098	42.8 (38.2 to 47.5)	29.2 (25.1 to 33.3)	4.6 (0.3 to 8.8)	1.19 (1.02 to 1.37)
**Sociodemographic characteristics**					
***Sex***					
Men	8852	22.7 (21.4 to 24.0)	23.8 (22.5 to 25.1)	Reference	Reference
Women	10,482	28.5 (27.3 to 29.7)	27.3 (26.2 to 28.5)	3.5 (2.1 to 5.0)	1.15 (1.08 to 1.22)
***Age (years)***					
18‐34	3574	13.8 (12.1 to 15.4)	13.4 (11.8 to 15.0)	Reference	Reference
35‐59	9748	21.8 (20.5 to 23.0)	21.5 (20.3 to 22.7)	8.1 (6.3 to 9.9)	1.60 (1.42 to 1.81)
60‐64	1798	38.0 (34.7 to 41.2)	38.6 (35.3 to 41.9)	25.2 (21.5 to 28.9)	2.87 (2.48 to 3.33)
65‐69	1831	48.4 (45.2 to 51.6)	49.2 (46.0 to 52.5)	35.8 (32.2 to 39.4)	3.66 (3.20 to 4.19)
70‐74	1183	45.9 (41.8 to 49.9)	47.1 (43.0 to 51.3)	33.7 (29.3 to 38.1)	3.51 (3.03 to 4.07)
75‐79	675	37.1 (32.3 to 41.8)	38.8 (33.9 to 43.8)	25.4 (20.2 to 30.6)	2.89 (2.43 to 3.44)
≥80	525	34.3 (28.7 to 40.0)	37.0 (30.9 to 43.1)	23.5 (17.2 to 29.9)	2.75 (2.24 to 3.38)
***Nationality***					
Spanish	18,692	26.1 (25.0 to 27.1)	25.9 (24.9 to 26.9)	Reference	Reference
Other	642	16.3 (12.6 to 20.1)	19.7 (15.5 to 23.9)	–6.2 (–10.4 to –2.0)	0.76 (0.61 to 0.94)
***Education***					
Primary or less	3858	32.6 (30.4 to 34.8)	23.4 (21.6 to 25.3)	Reference	Reference
High school/vocational education	11,250	22.9 (21.7 to 24.1)	24.8 (23.5 to 26.0)	1.3 (–0.8 to 3.4)	1.06 (0.97 to 1.15)
University	4226	27.3 (25.3 to 29.2)	30.0 (28.0 to 31.9)	6.5 (3.8 to 9.2)	1.28 (1.15 to 1.42)
**Household and residence characteristics**					
***Living with older adults***					
No	13,660	22.9 (21.8 to 24.0)	24.3 (23.2 to 25.4)	Reference	Reference
Yes	5671	32.9 (31.0 to 34.8)	29.0 (27.2 to 30.7)	4.7 (2.7 to 6.6)	1.19 (1.11 to 1.28)
***Household size***					
1‐2 people	6615	32.9 (31.2 to 34.7)	26.2 (24.7 to 27.8)	Reference	Reference
≥3 people	12,719	22.1 (21.0 to 23.2)	25.4 (24.1 to 26.6)	–0.9 (–2.8 to 1.1)	0.97 (0.90 to 1.04)
***Municipality size (population)***					
>100,000	5776	26.8 (25.2 to 28.4)	26.0 (24.5 to 27.5)	Reference	Reference
20,000‐100,000	5829	24.9 (23.0 to 26.9)	25.9 (24.0 to 27.8)	–0.1 (–2.6 to 2.3)	0.99 (0.91 to 1.09)
5000‐20,000	4167	25.1 (22.9 to 27.2)	25.4 (23.4 to 27.5)	–0.6 (–3.1 to 1.9)	0.98 (0.89 to 1.08)
<5000	3562	24.6 (21.8 to 27.3)	24.5 (21.3 to 27.6)	–1.6 (–5.1 to 2.0)	0.94 (0.82 to 1.08)
***Relative income of the residential area***					
Under 25th percentile	5226	25.4 (23.1 to 27.7)	27.1 (24.8 to 29.3)	Reference	Reference
25th to 50th percentile	4776	24.4 (22.7 to 26.2)	25.0 (23.2 to 26.8)	–2.1 (–5.0 to 0.8)	0.92 (0.83 to 1.03)
50th to 75th percentile	4424	24.2 (22.2 to 26.2)	23.9 (22.0 to 25.7)	–3.2 (–6.1 to –0.2)	0.88 (0.79 to 0.99)
Over 75th percentile	4908	28.8 (26.8 to 30.9)	26.9 (25.0 to 28.7)	–0.2 (–3.1 to 2.7)	0.99 (0.89 to 1.11)
**Health-related characteristics**					
***Self-rated health***					
Very good or good	17,029	24.9 (23.8 to 26.0)	25.4 (24.4 to 26.5)	Reference	Reference
Bad or very bad	2305	31.8 (29.3 to 34.2)	27.4 (25.2 to 29.6)	2.0 (–0.4 to 4.4)	1.08 (0.99 to 1.18)
***Chronic diseases***					
None	8740	19.8 (18.6 to 21.0)	22.9 (21.2 to 24.5)	Reference	Reference
1	5130	28.9 (27.2 to 30.7)	26.9 (25.2 to 28.6)	4.0 (1.6 to 6.5)	1.18 (1.07 to 1.30)
2‐3	4644	31.9 (29.9 to 33.8)	28.2 (26.1 to 30.2)	5.3 (2.5 to 8.2)	1.23 (1.10 to 1.38)
≥4	820	38.3 (33.9 to 42.7)	30.8 (26.8 to 34.9)	8.0 (3.4 to 12.5)	1.35 (1.15 to 1.58)
***Disability***					
No	18,184	25.3 (24.3 to 26.3)	25.5 (24.5 to 26.5)	Reference	Reference
Yes	1150	32.1 (28.5 to 35.7)	28.3 (24.9 to 31.8)	2.8 (–0.7 to 6.4)	1.11 (0.98 to 1.26)
***BMI***					
Underweight and normal weight	7879	23.4 (22.0 to 24.7)	25.1 (23.6 to 26.6)	Reference	Reference
Overweight	7351	26.4 (24.9 to 27.9)	25.2 (23.8 to 26.7)	0.1 (–1.8 to 2.1)	1.01 (0.93 to 1.09)
Obesity	4101	29.4 (27.4 to 31.3)	27.7 (25.8 to 29.6)	2.6 (0.3 to 4.9)	1.10 (1.01 to 1.20)
*** Tobacco use***					
Never	9584	25.6 (24.3 to 26.9)	25.5 (24.1 to 26.8)	Reference	Reference
Former	4903	32.8 (30.9 to 34.7)	28.8 (27.0 to 30.6)	3.4 (1.3 to 5.4)	1.13 (1.05 to 1.22)
Current	4847	19.3 (17.7 to 20.9)	22.7 (21.0 to 24.5)	–2.7 (–4.8 to –0.7)	0.89 (0.82 to 0.97)
**COVID-19–related characteristics**					
***Personal history***					
No infection	16,510	25.8 (24.8 to 26.9)	26.0 (24.9 to 27.0)	Reference	Reference
Not-severe infection	2361	23.5 (20.8 to 26.3)	23.2 (20.6 to 25.9)	–2.7 (–5.4 to 0.0)	0.89 (0.80 to 1.00)
Pneumonia or hospitalization	463	31.0 (24.8 to 37.1)	28.6 (22.9 to 34.3)	2.6 (–3.1 to 8.4)	1.10 (0.90 to 1.35)
***Contact with case***					
No contact	11,685	26.2 (24.9 to 27.4)	24.5 (23.3 to 25.7)	Reference	Reference
Noncohabitant symptomatic contact	974	24.1 (20.3 to 28.0)	26.4 (22.4 to 30.5)	1.9 (–2.2 to 6.1)	1.08 (0.92 to 1.26)
Noncohabitant case contact	3226	25.5 (23.3 to 27.7)	27.8 (25.6 to 30.0)	3.3 (0.9 to 5.8)	1.14 (1.04 to 1.24)
Household symptomatic contact	1035	23.7 (20.1 to 27.3)	27.4 (23.6 to 31.3)	2.9 (–1.0 to 6.9)	1.12 (0.97 to 1.29)
Household case contact	2414	25.3 (22.3 to 28.4)	27.5 (24.4 to 30.6)	3.0 (–0.2 to 6.2)	1.12 (1.00 to 1.26)
***Provincial 7-day cumulative incidence (per 100,000)***					
<100	1654	22.6 (19.8 to 25.4)	33.7 (27.3 to 40.1)	Reference	Reference
100‐200	12,249	25.8 (24.5 to 27.0)	25.4 (24.1 to 26.8)	–8.3 (–15.0 to –1.5)	0.75 (0.62 to 0.93)
200‐300	4925	26.5 (24.4 to 28.7)	24.2 (21.7 to 26.7)	–9.5 (–16.6 to –2.3)	0.72 (0.57 to 0.90)
≥300	506	27.0 (21.2 to 32.8)	26.6 (20.3 to 32.8)	–7.1 (–15.5 to 1.3)	0.79 (0.59 to 1.05)
***Face mask type used***					
Cloth	3207	18.0 (16.3 to 19.8)	20.9 (19.0 to 22.8)	Reference	Reference
Surgical	12,183	26.1 (24.9 to 27.3)	25.5 (24.3 to 26.7)	4.7 (2.5 to 6.8)	1.22 (1.11 to 1.35)
FFP2	3924	31.1 (29.0 to 33.3)	29.8 (27.8 to 31.9)	9.0 (6.2 to 11.7)	1.43 (1.28 to 1.60)
***Face mask use in family meetings***					
No	6898	21.9 (20.5 to 23.3)	22.9 (21.5 to 24.3)	Reference	Reference
Sometimes	1873	24.8 (22.0 to 27.6)	26.7 (23.9 to 29.6)	3.8 (0.7 to 7.0)	1.17 (1.03 to 1.32)
Yes	7634	27.8 (26.1 to 29.5)	27.3 (25.6 to 28.9)	4.4 (2.2 to 6.5)	1.19 (1.09 to 1.30)
Did not meet	2929	31.1 (28.6 to 33.6)	28.0 (25.7 to 30.2)	5.1 (2.5 to 7.6)	1.22 (1.11 to 1.35)
***Indoor bar attendance***					
<Once/month	13,812	27.0 (25.8 to 28.2)	26.0 (24.9 to 27.2)	Reference	Reference
1‐3 times/month	2980	23.0 (20.8 to 25.2)	25.2 (22.9 to 27.5)	–0.8 (–3.3 to 1.7)	0.97 (0.88 to 1.07)
≥Once/week	2542	21.8 (19.4 to 24.2)	24.2 (21.8 to 26.6)	–1.8 (–4.4 to 0.8)	0.93 (0.84 to 1.04)
*** Indoor family meetings with >10 people***					
Never	16,461	26.6 (25.5 to 27.7)	25.9 (24.9 to 27.0)	Reference	Reference
1‐5 times	2299	20.6 (18.4 to 22.9)	23.9 (21.5 to 26.3)	–2.0 (–4.6 to 0.5)	0.92 (0.83 to 1.02)
>5 times	574	21.7 (17.0 to 26.5)	25.1 (20.4 to 29.7)	–0.9 (–5.6 to 3.9)	0.97 (0.80 to 1.17)
***Indoor events, most people without mask***					
No	17,450	26.4 (25.3 to 27.4)	25.9 (24.8 to 26.9)	Reference	Reference
Yes	1884	19.9 (17.4 to 22.4)	23.9 (21.1 to 26.7)	–1.9 (–4.9 to 1.0)	0.93 (0.82 to 1.05)

a The 2020 campaign was not finished when the survey took place; in this table, participants answering that they had still not received the vaccine but intended to be vaccinated were considered vaccinated.

b Standardized to the overall distribution of influenza vaccination group, sex, age, education, and autonomous community in the unvaccinated target population in 2019, except for influenza vaccination groups and age groups that were not mutually standardized.

**Figure 3.**
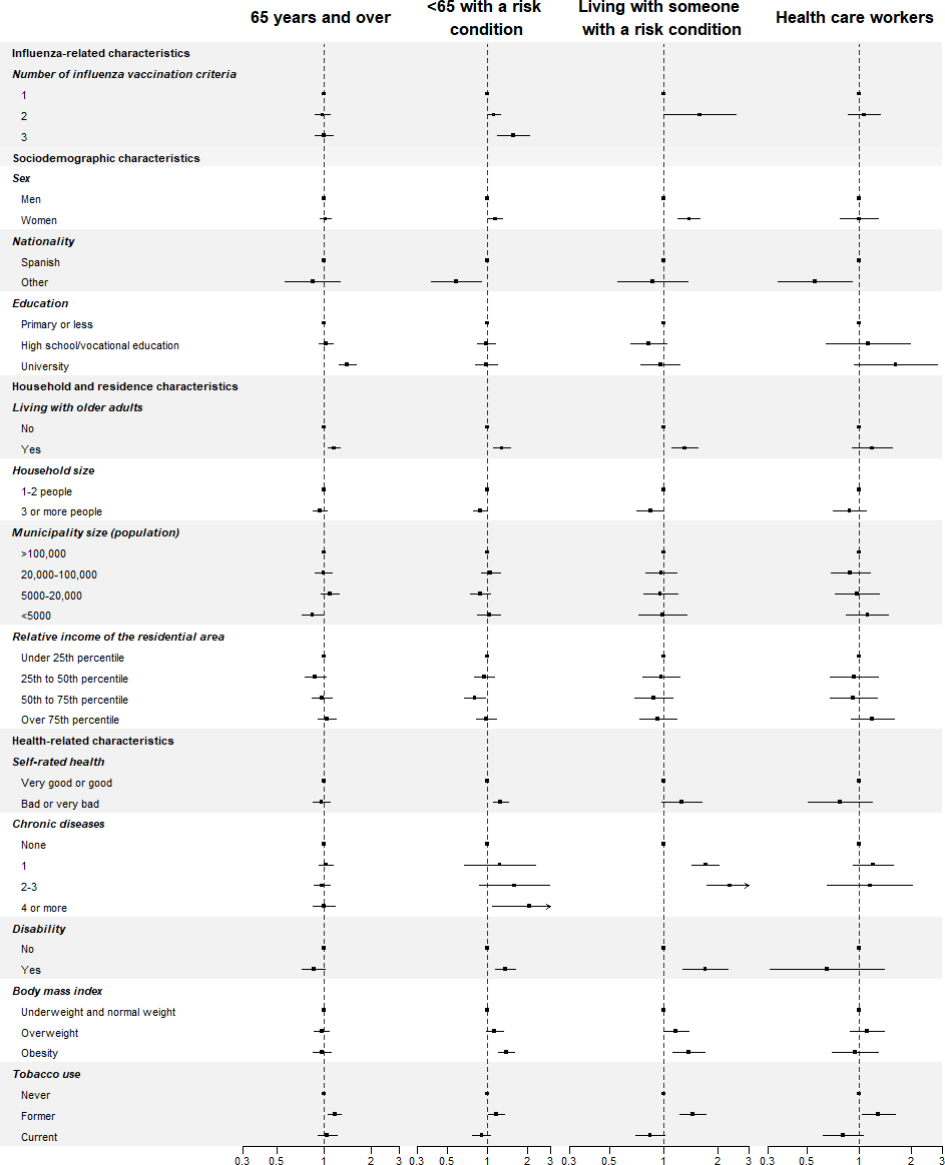
Standardized prevalence ratios of new influenza vaccination in 2020 among participants unvaccinated in 2019 across influenza-related, sociodemographic, and health-related factors, by target group.

**Figure 4.**
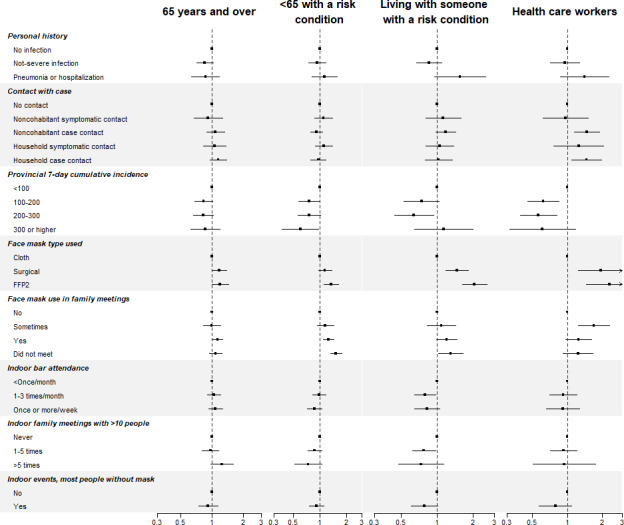
Standardized prevalence ratios of new influenza vaccination in 2020 among participants unvaccinated in 2019 across COVID-19–related factors, by target group.

Some of these associations differed by target group (Figure 3, Figure 4, Table S2 in Multimedia Appendix 1). Number of influenza vaccination criteria, female sex, and poorer health status were related to a higher prevalence of new vaccinees mainly in people younger than 65 years with risk conditions or those living with someone at risk (*P* heterogeneity ranged from <.001 to .01). In contrast, university education showed a higher proportion of new vaccinees in people older than 65 years (SPR: 1.41, 95% CI 1.24‐1.60) and in HCWs (SPR: 1.62, 0.94‐2.79; *P* heterogeneity=.003). With respect to COVID-19–related factors (Figure 4), contact with COVID-19 cases was associated with new vaccinations mainly in HCWs (*P* heterogeneity=.10). The higher prevalence of new influenza vaccine uptake among those wearing surgical and FFP2 face masks compared to cloth masks was stronger in HCWs and people living with someone with risk conditions (*P* heterogeneity=.003).

Sensitivity analyses excluding participants reporting their intention to be vaccinated in the 2020‐2021 campaign but who had not yet received the vaccine led to a 5% lower vaccination coverage estimate in 2020 and a 6% reduction in the absolute proportion of newly vaccinated. No remarkable differences were observed between both approaches in the reported associations with explanatory factors except for a slightly smaller increase in coverage in the group of individuals under 65 years old with risk conditions compared to the other target groups and a higher difference in the proportion of new vaccinees depending on the cumulative incidence in the province of residence (Tables S3-S4 in Multimedia Appendix 1).

## Discussion

### Principal Findings

One of the few positive side effects of the COVID-19 pandemic may have been the increase in the uptake of influenza vaccination, probably influenced by a higher awareness of the risk associated with respiratory diseases and the desire to avoid an infection that might be mistaken for COVID-19 and avoid any new saturation of the health care system. In this study, taking advantage of the ENE-COVID study design, we provide nationally representative estimates and a description of factors associated with influenza vaccination coverage in the main target groups in Spain in the influenza season before the appearance of SARS-CoV-2 (2019/2020) and compare it to the first season after its emergence (2020/2021). Our data show a generalized increase in vaccination, especially among HCWs. In addition, we showed that among people unvaccinated in 2019, older people, women, more educated groups, those with comorbidities, or those living with older adults were more prone to accept the vaccine in 2020. Some COVID-19–related factors were also found to be associated with the decision to vaccinate against seasonal influenza, such as the perceived risk of SARS-CoV-2 infection, reflected here by the awareness of having had contact with COVID-19 cases, the type of masks used, their use in family meetings, and not attending family/friend meetings. It should be noted that this study was conducted in November 2020, when the influenza vaccination campaign was ongoing, the number of COVID-19 cases was quickly rising (the third COVID-19 epidemic wave), COVID-19 vaccines were still not authorized, and more than 40,000 COVID-19–related deaths had occurred in the country.

During the 2020/2021 influenza season, a historical reduction in influenza transmission was observed worldwide, including in Spain [19,20]. The circulation of the highly transmissible SARS-CoV-2 in a fully susceptible population probably displaced other respiratory viruses, including influenza, which, together with COVID-19 mitigation measures, probably had a relevant role, further aided by the increase in influenza vaccination. Fear of unknown but potentially devastating effects of a “twindemic” [21,22] may have favored the adoption of preventive behaviors, including influenza vaccination, as suggested in other countries [14,23,24]. The reinforced public health influenza vaccination campaign implemented that year, with specific messages targeted at HCWs, likely contributed to increased coverage. Our data allowed us to quantify this effect, showing that, in Spain, vaccination coverage among the main target groups augmented from 31.4% in 2019 to 46.8% in 2020, breaking the downward trend observed in Spain [15,25] and other European countries in previous years [3].

### Comparison With Prior Work

In 2020/2021, coverage was around 75% among older people, attaining the recommended goal established by the World Health Organization and the European Council [5]. This figure is higher than the 66% published by the Spanish Ministry of Health [15], although they are not fully comparable, as they come from different data sources (self-reported versus regional health information systems) and there are 2 regions not included in the estimation provided by the Ministry. For the other target groups, even though still remaining far from the goal [5,15], coverage figures also showed a marked improvement, which was particularly noteworthy among HCWs, a group heavily affected by the pandemic that year. The burden experienced during the first COVID-19 epidemic wave, when the health care system in Spain became overwhelmed, could have boosted this practice, though it was not maintained in 2021 and 2022 [15]. This finding, also observed in other European countries [26], contrasts with the stable evolution in the 65 years and older population, which may reflect the success of the vaccination strategy for this age group, which included the simultaneous administration of the influenza vaccine and the COVID-19 booster in a context of high COVID-19 vaccine effectiveness. On the other hand, among HCWs, risk perception may have decreased once effective COVID-19 vaccines became available, reducing their willingness to receive the influenza vaccine. Still, the drop in coverage did not reach the low levels seen before the pandemic [27]. HCWs reported that important reasons driving influenza vaccine uptake in 2020/2021 were self-protection, protecting family, and protecting patients [26,28]. Highlighting these benefits of vaccination in campaigns aimed at promoting influenza vaccine uptake among HCWs may contribute to their effectiveness, which is especially relevant, as vaccination is a basic measure to protect at-risk people that HCWs work closely with and HCWs’ advice may influence the decisions of others.

Focusing on new vaccinations, 1 of 4 people in target groups that were unvaccinated in 2019 reported vaccination in 2020. This figure is similar to estimations based on vaccination intention in the early months of the pandemic [29,30] and is consistent with the results of an Italian survey [14], where 20.4% of adults reported that their intention to receive the influenza vaccination in 2020/2021 was due to the COVID-19 pandemic. Our data showed that the increase in vaccine uptake was not evenly distributed. New vaccination was more frequent in older adults, in women, among Spaniards, and among those with higher education, which is consistent with findings in the prepandemic literature [31]. In addition, current smokers were less likely to change to receiving the vaccine, which could reflect generally lower engagement with healthy lifestyles. Regarding household composition, living with older people was associated with higher vaccination uptake in 2020. Households are an important setting for the transmission of respiratory diseases, with risk of infection from a household contact being up to 38% [32,33].

Pandemic-related factors, such as worries about COVID-19, have been described in the literature as being associated with the intention to receive the influenza vaccine [34]. Our results showed that people who might be considered more cautious, as reflected by their reported attitude toward the prevention of SARS-CoV-2 transmission, showed higher probability of being newly vaccinated. These prudent behaviors may have been prompted by what has been called cues to action, which include both internal and environmental triggers that may activate people’s motivation to engage in healthy activities [30]. Overall, these findings also suggest that both protection of others and self-protection represent motivations for vaccination. HCWs are a clear example of this [26,28]. In our study, contact with confirmed COVID-19 cases showed a stronger association with opting for influenza vaccination in 2020 among HCWs than in other target groups.

One of the main strengths of this study is the use of data from the nationwide population-based ENE-COVID study, based on a big sample representative of the noninstitutionalized population in Spain. The high participation rate, as well as the poststratification weights used in the analyses, allowed us to diminish the risk of bias in our estimations. We have analyzed many factors, several of them not usually available at a national level, potentially related to influenza vaccination in people from 5 target groups, allowing for a deeper understanding of the determinants of influenza vaccination. In addition, we evaluated factors associated with the change in influenza vaccine uptake just after the pandemic and provided standardized estimates that took into account possible confounders.

### Limitations

This study also had some limitations. First, people living in nursing homes (around 4% of the population aged 65 years and older in Spain) [35] were not included in the ENE-COVID study; consequently, our prevalence figures might be slightly underestimated, as vaccination among institutionalized older people is generally higher. Second, potential inaccuracies in the population register could have precluded contact with current residents in some of the selected households, potentially introducing some degree of selection bias. Additionally, most of the information was self-reported and may have been subject to recall and social desirability biases. In addition, data were collected before the 2020/2021 influenza vaccination campaign had finished (it was extended from the first week of October 2020 to the end of January 2021) and, therefore, for the 2020/2021 influenza season, some participants (7% of the sample) reported intention to be vaccinated rather than vaccination, which could overestimate vaccination rates. However, intention has been considered as a good proxy for vaccine uptake [34] and our sensitivity analysis excluding participants that intended to receive the vaccine showed similar associations with the studied factors. The standardized method used, based on logistic models, assumed no relevant effect modification in the exposure odds ratio by strata of the confounding factors. Therefore, we cannot rule out some degree of model misspecification, but we expect this method to provide stable results and leave little residual confounding. Regarding classification of participants into the target groups of people younger than 65 years with a risk condition or those living with someone with a risk condition, the questionnaire did not collect information on all pathologies that are included as risk conditions for influenza vaccination (ie, neurological diseases, nonmalignant blood diseases, asplenia, causes of immunodepression other than pharmacological treatments, and celiac and inflammatory bowel diseases). Likewise, we were not able to analyze some target groups, such as pregnant women. Another limitation is that we did not consider possible regional differences in changes in coverage and the factors associated with this change, thus limiting the direct extrapolation of our findings to the regional level. Finally, our results should be interpreted in the context of the Spanish National Health System, where influenza vaccination is free of charge for people in the recommended groups, which may reduce their generalizability to contexts with a different health care system. Future studies based on vaccination registries combined with clinical or self-reported information or carried out in specific settings (eg, nursing homes) could overcome some of these limitations.

### Conclusions

A large increase in influenza vaccination coverage was observed between 2019 and 2020 in the main target groups in Spain, particularly in HCWs, although vaccination goals were only attained in the group of people aged ≥65 years. In this group, this historic rise in coverage broke the declining trend observed in the previous years. However, now that the COVID-19 pandemic is under control, sustaining or improving these figures may prove challenging. Identifying the components of the 2020 reinforced influenza vaccination campaign that contributed to its effectiveness can inform future campaigns. Additionally, the presented crude and standardized estimates of the characteristics of the population not vaccinated in the past that were associated with becoming vaccinated in a posterior campaign may represent valuable insights to guide targeted interventions and vaccination strategies.

## Supplementary material

10.2196/60658 Multimedia Appendix 1 Tables S1-S4

## Abbreviations

ENE-COVID: Seroepidemiological Survey of SARS-CoV-2 Infection in Spain
HCW: health care worker
SPR: standardized prevalence ratio
