# Supplementary material for: Influenza Vaccination Coverage and Determinants of New Vaccinations During the COVID-19 Pandemic in Spain (ENE-COVID): Nationwide Population-Based Study
Source: JMIR Public Health Surveill. 2025 Jul 1;11:e60658. doi: 10.2196/60658 (PMC12236637; doi:10.2196/60658)
Supplement: Multimedia Appendix 1 [file publichealth-v11-e60658-s001.pdf]

Multimedia Appendix 1

Supplement to: de la Cámara MA, Fernández de Larrea-Baz N, Pastor-Barriuso R, et al. Influenza vaccination coverage and determinants of new vaccinations during the COVID-19 pandemic in Spain (ENE-COVID): Nationwide population-based study. JMIR Public Health and Surveillance.

Contents

Table S1. Cross-classified prevalences of influenza vaccination in 2019 and 2020 by sociodemographic, health and COVID-19 epidemic-related characteristics among adults pertaining to selected influenza vaccination target groups (ENE-COVID study, Spain, November 2020). 2

Table S2. Standardized prevalence ratio of new influenza vaccination in 2020[a] among participants unvaccinated in 2019 across influenza-related, sociodemographic, health-related, and COVID-19-related factors, by target group. 4

Table S3. Influenza vaccination coverage in 2019 and 2020[a] and coverage differences among adults of selected vaccination target groups[b], excluding participants that intended but had still not received the 2020 vaccine (ENE-COVID study, Spain, November 2020). 6

Table S4. Prevalence of new influenza vaccination in 2020[a] among participants unvaccinated in 2019, by sociodemographic, health-related, and COVID-19-related factors, excluding those that intended but had still not received the 2020 vaccine (ENE-COVID study). 8

Table S1. Cross-classified prevalences of influenza vaccination in 2019 and 2020 by sociodemographic, health and COVID-19 epidemic-related characteristics among adults pertaining to selected influenza vaccination target groups (ENE-COVID study, Spain, November 2020).

		None		Only in 2019		Both years		Only in 2020
	N	% (95% CI)[a]	N	% (95% CI)[a]	N	% (95% CI)[a]	N	% (95% CI)[a]
Total	14,244	51.0 (50.0-52.0)	639	2.2 (1.9-2.5)	8851	29.2 (28.3-30.0)	5253	17.6 (16.9-18.3)
Influenza related characteristics								
**Influenza vaccination target group**								
≥65 years	2382	23.3 (22.0-24.6)	197	1.9 (1.5-2.3)	5841	56.4 (54.9-58.0)	1859	18.4 (17.2-19.5)
<65 with a risk condition	4350	55.8 (54.2-57.4)	209	2.8 (2.3-3.4)	1859	22.2 (20.9-23.4)	1582	19.2 (17.9-20.5)
Health care workers	1074	52.2 (49.0-55.4)	58	2.5 (1.6-3.4)	484	20.8 (18.2-23.4)	556	24.5 (21.9-27.1)
Living with someone with a risk condition	6185	77.1 (75.7-78.5)	162	1.8 (1.4-2.2)	649	7.4 (6.6-8.3)	1194	13.6 (12.5-14.7)
Security and emergency workers	253	75.0 (68.8-81.2)	13	4.0 (1.2-6.8)	18	4.6 (1.8-7.4)	62	16.4 (11.1-21.8)
**Number of influenza vaccination criteria**								
1	10,141	53.5 (52.2-54.8)	373	2.3 (1.9-2.7)	3085	25.7 (24.5-26.8)	2984	18.5 (17.6-19.5)
2	3479	49.5 (47.9-51.1)	216	2.2 (1.8-2.6)	3836	30.4 (29.1-31.7)	1790	17.9 (16.6-19.1)
3	624	43.3 (40.1-46.6)	50	1.9 (1.1-2.6)	1930	38.7 (36.2-41.2)	479	16.1 (13.8-18.4)
Sociodemographic characteristics								
**Sex**								
Men	6776	52.8 (51.5-54.0)	273	2.1 (1.7-2.4)	3975	28.9 (27.9-29.9)	2161	16.3 (15.4-17.2)
Women	7468	49.5 (48.3-50.6)	366	2.3 (2.0-2.7)	4876	29.5 (28.4-30.5)	3092	18.7 (17.9-19.6)
**Age**								
18-34	3115	77.1 (75.3-78.9)	124	2.7 (2.0-3.4)	318	8.0 (6.8-9.2)	502	12.2 (10.8-13.6)
35-59	7618	64.8 (63.5-66.1)	261	2.2 (1.8-2.6)	1828	14.9 (14.0-15.8)	2213	18.1 (17.1-19.1)
60-64	1129	40.5 (37.8-43.1)	57	2.4 (1.6-3.2)	864	32.3 (29.8-34.8)	679	24.9 (22.5-27.2)
65-69	970	27.5 (25.4-29.6)	57	1.7 (1.1-2.2)	1439	45.1 (42.7-47.6)	873	25.7 (23.6-27.8)
70-74	641	23.8 (21.4-26.1)	54	1.9 (1.3-2.6)	1554	54.2 (51.4-56.9)	550	20.1 (18.0-22.2)
75-79	422	22.1 (19.6-24.6)	44	2.1 (1.2-3.0)	1265	62.8 (59.9-65.7)	257	13.1 (11.2-14.9)
≥80	349	17.1 (14.8-19.3)	42	2.0 (1.2-2.7)	1583	72.1 (69.2-74.9)	179	8.9 (7.1-10.7)
**Nationality**								
Spanish	13,697	50.7 (49.7-51.7)	609	2.2 (1.9-2.4)	8722	29.4 (28.5-30.3)	5144	17.8 (17.1-18.5)
Other	547	61.9 (57.6-66.3)	30	3.0 (1.6-4.4)	129	20.9 (17.0-24.8)	109	14.1 (10.9-17.3)
**Education**								
Primary or less	2619	49.3 (47.6-51.0)	176	2.3 (1.8-2.7)	3796	32.7 (31.3-34.2)	1239	15.7 (14.5-17.0)
High school/vocational education	8550	53.3 (52.0-54.5)	349	2.2 (1.9-2.6)	3592	26.8 (25.7-27.9)	2700	17.7 (16.8-18.6)
University	2955	48.0 (46.2-49.8)	111	2.2 (1.6-2.7)	1408	29.6 (27.7-31.4)	1271	20.2 (18.7-21.8)
**Employment status**								
Actively working	7599	57.9 (56.1-59.7)	238	2.0 (1.6-2.4)	1714	22.0 (20.4-23.6)	2179	18.1 (16.8-19.4)
Student	775	68.5 (63.5-73.5)	24	1.9 (0.9-2.8)	75	17.7 (12.7-22.7)	100	12.0 (8.8-15.1)
Retired	2702	40.5 (38.6-42.5)	212	2.6 (1.8-3.4)	5765	36.5 (34.9-38.2)	1957	20.3 (18.6-22.0)
Unemployed/other	3168	52.8 (50.9-54.7)	165	2.7 (2.2-3.3)	1297	26.8 (25.2-28.4)	1017	17.7 (16.2-19.2)
**On-site activity**								
No	5914	47.0 (45.8-48.3)	367	2.5 (2.0-2.9)	7024	32.2 (31.1-33.4)	2962	18.3 (17.3-19.3)
On-site work	7019	55.3 (53.7-57.0)	215	1.9 (1.5-2.3)	1578	24.1 (22.5-25.6)	2035	18.7 (17.5-19.9)
Other on-site activity	1311	61.1 (57.7-64.4)	57	2.9 (1.8-3.9)	249	22.3 (19.1-25.4)	256	13.8 (11.6-15.9)
Household and residence characteristics								
**Living with older adults**								
No	10,432	53.3 (52.2-54.4)	436	2.2 (1.9-2.5)	4550	27.4 (26.4-28.4)	3360	17.1 (16.3-17.9)
Yes	3809	46.3 (44.7-48.0)	203	2.2 (1.8-2.7)	4290	32.2 (30.8-33.6)	1893	19.3 (18.1-20.4)
**Living with children <3 years**								
No	13,558	51.1 (50.1-52.1)	594	2.1 (1.9-2.4)	8610	29.2 (28.3-30.1)	5030	17.6 (16.9-18.3)
Yes	686	49.3 (45.4-53.3)	45	4.0 (2.5-5.5)	241	29.2 (25.1-33.2)	223	17.5 (14.4-20.7)
**Household size**								
1-2 people	4425	49.2 (47.8-50.6)	242	2.3 (1.9-2.7)	4899	30.0 (28.8-31.2)	2250	18.5 (17.4-19.6)
≥3 people	9819	52.2 (50.9-53.4)	397	2.1 (1.8-2.5)	3952	28.5 (27.3-29.7)	3003	17.2 (16.3-18.1)
**Municipality size (population)**								
>100,000	4165	50.1 (48.5-51.8)	198	2.3 (1.8-2.7)	2706	29.7 (28.3-31.1)	1658	17.9 (16.9-19.0)
20,000-100,000	4317	51.7 (50.0-53.4)	189	2.2 (1.7-2.7)	2411	28.3 (26.9-29.7)	1578	17.7 (16.4-19.1)
5000-20,000	3149	52.0 (50.2-53.8)	124	2.0 (1.5-2.5)	1751	28.2 (26.5-29.9)	1051	17.8 (16.3-19.4)
<5000	2613	51.0 (48.5-53.4)	128	2.2 (1.6-2.9)	1983	30.7 (29.0-32.4)	966	16.1 (14.2-18.0)
**Relative income of the residential area**								
Under p25	3907	51.6 (49.6-53.5)	189	2.3 (1.8-2.7)	2301	28.3 (26.9-29.8)	1360	17.8 (16.2-19.4)
P25-50	3561	50.6 (48.7-52.4)	156	2.2 (1.7-2.8)	2298	30.8 (29.2-32.5)	1257	16.4 (15.2-17.6)
P50-75	3334	53.0 (51.2-54.8)	149	2.3 (1.8-2.9)	1914	27.6 (26.2-29.1)	1120	17.0 (15.6-18.4)
Over p75	3442	49.0 (47.1-50.9)	145	1.9 (1.4-2.4)	2338	29.8 (28.0-31.6)	1516	19.3 (17.9-20.6)

	None		Only in 2019		Both years		Only in 2020	
	N	% (95% CI)[a]	N	% (95% CI)[a]	N	% (95% CI)[a]	N	% (95% CI)[a]
Health-related characteristics								
**Self-rated health**								
Very good or good	12,661	52.1 (51.1-53.2)	512	2.1 (1.9-2.4)	6764	27.8 (26.9-28.7)	4514	17.9 (17.1-18.7)
Bad or very bad	1583	44.9 (43.0-46.8)	127	2.5 (1.9-3.1)	2087	35.9 (34.2-37.5)	739	16.7 (15.2-18.1)
**Chronic diseases**								
None	6956	59.4 (57.8-61.0)	231	2.3 (1.8-2.9)	1657	20.2 (18.8-21.6)	1876	18.1 (16.7-19.4)
1	3626	50.7 (49.1-52.3)	174	2.4 (1.9-2.9)	2312	27.6 (26.3-28.9)	1534	19.3 (18.1-20.6)
2-3	3141	44.9 (43.2-46.7)	176	1.9 (1.5-2.4)	3595	35.3 (33.8-36.8)	1539	17.8 (16.6-19.1)
≥4	521	34.9 (32.0-37.8)	58	2.8 (1.6-3.9)	1287	47.2 (44.4-50.0)	304	15.1 (13.0-17.3)
**Disability**								
No	13,389	51.6 (50.6-52.7)	598	2.2 (1.9-2.5)	7875	28.5 (27.6-29.4)	4826	17.6 (16.9-18.4)
Yes	760	42.7 (39.8-45.5)	38	1.9 (1.1-2.7)	930	37.8 (35.2-40.4)	390	17.6 (15.4-19.7)
**Body mass index**								
Underweight and normal weight	5977	52.9 (51.5-54.3)	232	2.2 (1.8-2.6)	2815	27.5 (26.3-28.6)	1962	17.5 (16.4-18.5)
Overweight	5349	50.8 (49.4-52.2)	230	2.0 (1.6-2.3)	3779	29.8 (28.6-30.9)	2067	17.4 (16.4-18.4)
Obesity	2915	48.0 (46.4-49.7)	176	2.6 (2.0-3.1)	2256	31.0 (29.5-32.4)	1224	18.4 (17.1-19.7)
**Tobacco use**								
Never	7085	51.0 (49.7-52.3)	339	2.3 (1.9-2.6)	4851	29.7 (28.6-30.8)	2573	17.0 (16.1-17.9)
Former	3255	45.7 (44.1-47.2)	153	1.9 (1.5-2.3)	2986	32.5 (31.1-33.8)	1679	20.0 (18.8-21.2)
Current	3904	58.0 (56.2-59.7)	147	2.5 (2.0-3.0)	1012	22.5 (21.0-24.0)	1001	17.0 (15.6-18.4)
COVID-19-related								
**Personal history**								
No infection	12,134	50.8 (49.8-51.8)	543	2.2 (1.9-2.5)	7602	29.3 (28.4-30.2)	4521	17.7 (17.0-18.4)
Not-severe infection	1782	53.3 (50.8-55.9)	76	2.0 (1.4-2.6)	996	28.3 (26.2-30.4)	591	16.4 (14.5-18.3)
Pneumonia or hospitalization	328	47.7 (43.0-52.5)	20	3.1 (1.4-4.9)	253	28.9 (25.3-32.5)	141	20.2 (16.0-24.5)
**Contact with case**								
No contact	8577	51.3 (50.1-52.5)	393	2.2 (1.9-2.5)	6392	29.7 (28.7-30.6)	3222	16.9 (16.1-17.7)
Noncohabitant symptomatic contact	737	53.9 (50.4-57.5)	27	1.7 (0.9-2.4)	249	24.7 (21.6-27.9)	241	19.7 (16.4-22.9)
Noncohabitant case contact	2343	50.3 (48.4-52.2)	92	2.1 (1.6-2.7)	982	28.3 (26.3-30.3)	908	19.2 (17.4-21.0)
Household symptomatic contact	790	51.4 (48.0-54.8)	36	2.4 (1.3-3.4)	302	27.5 (23.8-31.1)	252	18.8 (15.8-21.8)
Household case contact	1797	49.8 (47.1-52.6)	91	2.4 (1.7-3.1)	926	29.5 (27.2-31.8)	630	18.3 (16.1-20.5)
**Cumulative provincial 7-day incidence (per 100,000)**								
<100	1269	55.7 (52.9-58.5)	61	2.5 (1.7-3.3)	597	25.0 (22.8-27.2)	404	16.8 (14.9-18.8)
100-200	9081	50.5 (49.3-51.6)	421	2.2 (1.9-2.5)	5611	29.8 (28.8-30.9)	3265	17.5 (16.7-18.4)
200-300	3531	51.4 (49.3-53.5)	139	2.1 (1.5-2.7)	2336	28.1 (26.4-29.8)	1441	18.4 (16.9-19.8)
≥300	363	48.3 (43.7-52.8)	18	1.9 (1.0-2.9)	307	33.1 (29.4-36.9)	143	16.7 (13.1-20.2)
**Number of people in close contact**								
None	1272	50.7 (48.0-53.3)	65	2.4 (1.4-3.5)	1179	31.2 (28.8-33.6)	504	15.7 (13.6-17.8)
1-2	3576	48.9 (47.3-50.5)	199	2.4 (1.9-2.9)	3345	30.0 (28.7-31.3)	1668	18.8 (17.5-20.1)
3-5	5970	50.9 (49.6-52.3)	255	2.1 (1.7-2.4)	3088	29.4 (28.1-30.6)	2059	17.6 (16.6-18.6)
6-9	2438	52.4 (50.4-54.4)	95	2.3 (1.7-2.9)	992	26.7 (24.9-28.5)	803	18.7 (17.0-20.3)
≥10	988	58.8 (55.3-62.2)	25	1.7 (0.5-2.8)	247	25.7 (22.4-28.9)	219	13.9 (11.5-16.3)
**Face mask type used**								
Cloth	2581	57.1 (55.2-58.9)	101	2.4 (1.8-3.0)	975	26.1 (24.3-27.8)	655	14.5 (13.0-15.9)
Surgical	8975	51.5 (50.3-52.6)	386	2.1 (1.8-2.4)	5775	28.7 (27.7-29.7)	3313	17.7 (16.9-18.6)
FFP2	2675	45.6 (43.8-47.3)	146	2.3 (1.8-2.8)	2071	32.4 (30.9-34.0)	1278	19.7 (18.3-21.2)
**Face mask use in family meetings**								
No	5286	54.8 (53.4-56.2)	226	2.2 (1.8-2.7)	2669	26.9 (25.7-28.2)	1669	16.1 (15.0-17.1)
Sometimes	1405	51.5 (49.0-53.9)	63	2.4 (1.7-3.2)	668	27.8 (25.6-29.9)	482	18.3 (16.2-20.4)
Yes	5501	48.7 (47.2-50.1)	250	2.1 (1.7-2.5)	3912	31.1 (29.8-32.3)	2189	18.2 (17.0-19.3)
Did not meet	2052	47.8 (45.8-49.8)	100	2.2 (1.6-2.8)	1602	30.3 (28.5-32.0)	913	19.7 (18.1-21.4)
**Indoor bar attendance**								
<Once/month	9978	49.8 (48.7-51.0)	497	2.3 (2.0-2.6)	7196	30.2 (29.2-31.1)	3958	17.7 (16.9-18.5)
1-3 times/month	2293	54.2 (52.2-56.3)	71	1.9 (1.3-2.6)	867	25.7 (23.9-27.5)	707	18.1 (16.4-19.9)
≥Once/week	1973	54.6 (52.3-57.0)	71	1.9 (1.3-2.5)	788	26.4 (24.3-28.6)	588	17.0 (15.2-18.9)
**Indoor family meetings with >10 people**								
Never	12,008	50.5 (49.5-51.5)	540	2.2 (1.9-2.5)	7941	29.5 (28.6-30.4)	4589	17.8 (17.1-18.6)
1-5 times	1785	54.6 (52.2-57.0)	72	2.1 (1.4-2.8)	706	26.7 (24.4-29.0)	535	16.6 (14.8-18.4)
>5 times	451	52.9 (48.5-57.2)	27	2.8 (1.4-4.1)	204	27.9 (23.7-32.1)	129	16.5 (12.9-20.1)
**Indoor events, most people without mask**								
No	12,753	50.6 (49.6-51.7)	577	2.2 (1.9-2.5)	8312	29.4 (28.5-30.3)	4840	17.8 (17.1-18.5)
Yes	1491	54.7 (52.2-57.3)	62	2.4 (1.6-3.2)	539	27.0 (24.5-29.4)	413	15.9 (13.9-17.9)
**Use of public transport**								
No	11,732	50.4 (49.3-51.4)	517	2.2 (1.9-2.5)	7453	29.6 (28.7-30.5)	4343	17.8 (17.0-18.6)
Yes	2512	53.4 (51.4-55.4)	122	2.3 (1.7-2.8)	1398	27.5 (25.8-29.2)	910	16.8 (15.4-18.2)

[a]Standardized to the overall distribution of influenza vaccination groups in the whole target population, except prevalences for age groups that were not standardized.

Table S2. Standardized prevalence ratio of new influenza vaccination in 2020[a] among participants unvaccinated in 2019 across influenza-related, sociodemographic, health-related, and COVID-19-related factors, by target group.

	65 years and over	<65y with a risk condition[b]	Living with somenone with a risk condition	Health care workers
	SPR (95% CI)	SPR (95% CI)	SPR (95% CI)	SPR (95% CI)
Influenza-related characteristics				
**Number of influenza vaccination criteria**				
1	Ref.	Ref.	Ref.	Ref.
2	0.97 (0.87-1.09)	1.13 (1.00-1.27)	1.59 (1.00-2.53)	1.07 (0.87-1.31)
3	1.00 (0.87-1.14)	1.57 (1.19-2.07)	-	-
Sociodemographic characteristics				
**Sex**				
Men	Ref.	Ref.	Ref.	Ref.
Women	1.02 (0.95-1.10)	1.15 (1.03-1.29)	1.39 (1.21-1.60)	1.00 (0.78-1.29)
**Nationality**				
Spanish	Ref.	Ref.	Ref.	Ref.
Other	0.84 (0.56-1.27)	0.59 (0.38-0.91)	0.87 (0.56-1.37)	0.56 (0.34-0.91)
**Education**				
Primary or less	Ref.	Ref.	Ref.	Ref.
High school/vocational education	1.03 (0.92-1.14)	0.99 (0.84-1.15)	0.83 (0.66-1.04)	1.12 (0.65-1.95)
University	1.41 (1.24-1.60)	0.99 (0.81-1.19)	0.96 (0.75-1.24)	1.62 (0.94-2.79)
Household and residence characteristics				
**Living with older adults**				
No	Ref.	Ref.	Ref.	Ref.
Yes	1.16 (1.05-1.27)	1.29 (1.13-1.49)	1.31 (1.11-1.54)	1.19 (0.91-1.55)
**Household size**				
1-2 people	Ref.	Ref.	Ref.	Ref.
3 or more people	0.94 (0.84-1.05)	0.89 (0.79-1.00)	0.85 (0.71-1.01)	0.88 (0.71-1.10)
**Municipality size (population)**				
>100,000	Ref.	Ref.	Ref.	Ref.
20,000-100,000	0.99 (0.87-1.13)	1.07 (0.91-1.25)	0.97 (0.80-1.18)	0.89 (0.69-1.15)
5000-20,000	1.09 (0.96-1.24)	0.89 (0.74-1.06)	0.96 (0.77-1.19)	0.97 (0.72-1.31)
<5000	0.84 (0.72-0.99)	1.04 (0.85-1.27)	0.99 (0.72-1.36)	1.12 (0.85-1.47)
**Relative income of the residential area**				
Under p25	Ref.	Ref.	Ref.	Ref.
P25-50	0.88 (0.75-1.02)	0.95 (0.80-1.14)	0.97 (0.77-1.23)	0.93 (0.68-1.28)
P50-75	0.97 (0.83-1.13)	0.81 (0.67-0.98)	0.88 (0.69-1.12)	0.93 (0.68-1.27)
Over p75	1.04 (0.91-1.20)	0.99 (0.83-1.19)	0.93 (0.73-1.18)	1.19 (0.90-1.58)
Health-related characteristics				
**Self-rated health**				
Very good or good	Ref.	Ref.	Ref.	Ref.
Bad or very bad	0.96 (0.85-1.09)	1.27 (1.11-1.45)	1.26 (0.97-1.63)	0.78 (0.51-1.19)
**Chronic diseases**				
None	Ref.	Ref.	Ref.	Ref.
1	1.03 (0.93-1.14)	1.25 (0.67-2.31)	1.71 (1.44-2.03)	1.20 (0.92-1.56)
2-3	0.97 (0.86-1.09)	1.61 (0.87-2.97)	2.34 (1.74-3.14)	1.15 (0.65-2.03)
4 or more	0.99 (0.84-1.17)	2.09 (1.10-3.96)	-	-
**Disability**				
No	Ref.	Ref.	Ref.	Ref.
Yes	0.86 (0.72-1.02)	1.38 (1.16-1.63)	1.71 (1.28-2.29)	0.66 (0.31-1.40)
**Body mass index**				
Underweight and normal weight	Ref.	Ref.	Ref.	Ref.
Overweight	0.96 (0.86-1.07)	1.14 (0.99-1.32)	1.17 (1.00-1.38)	1.11 (0.89-1.40)
Obesity	0.97 (0.85-1.10)	1.40 (1.21-1.61)	1.38 (1.13-1.69)	0.95 (0.70-1.29)
**Tobacco use**				
Never	Ref.	Ref.	Ref.	Ref.
Former	1.17 (1.06-1.28)	1.18 (1.03-1.34)	1.45 (1.23-1.71)	1.29 (1.03-1.61)
Current	1.05 (0.91-1.20)	0.91 (0.78-1.07)	0.84 (0.70-1.02)	0.81 (0.62-1.06)
COVID-19-related				
**Personal history**				
No infection	Ref.	Ref.	Ref.	Ref.
Not-severe infection	0.86 (0.71-1.03)	0.93 (0.74-1.16)	0.86 (0.67-1.09)	0.95 (0.71-1.27)
Pneumonia or hospitalization	0.87 (0.64-1.19)	1.13 (0.82-1.55)	1.55 (0.95-2.54)	1.41 (0.87-2.30)
**Contact with case**				
No contact	Ref.	Ref.	Ref.	Ref.
Noncohabitant symptomatic contact	0.92 (0.67-1.27)	1.10 (0.87-1.39)	1.12 (0.80-1.57)	0.97 (0.62-1.52)
Noncohabitant case contact	1.09 (0.89-1.32)	0.91 (0.78-1.06)	1.18 (0.97-1.42)	1.48 (1.15-1.90)
Household symptomatic contact	1.07 (0.83-1.37)	1.11 (0.89-1.39)	1.05 (0.80-1.38)	1.25 (0.76-2.07)
Household case contact	1.16 (0.95-1.40)	0.96 (0.79-1.17)	1.03 (0.79-1.34)	1.47 (1.08-1.99)

	65 years and over	<65y with a risk condition[b]	Living with somenone with a risk condition	Health care workers
	SPR (95% CI)	SPR (95% CI)	SPR (95% CI)	SPR (95% CI)
**Cumulative provincial 7-day incidence (per 100,000)**				
<100	Ref.	Ref.	Ref.	Ref.
100-200	0.83 (0.68-1.02)	0.75 (0.58-0.99)	0.74 (0.53-1.04)	0.62 (0.46-0.84)
200-300	0.83 (0.66-1.04)	0.76 (0.56-1.02)	0.64 (0.44-0.93)	0.56 (0.39-0.81)
300 or higher	0.87 (0.63-1.21)	0.60 (0.37-0.96)	1.14 (0.65-2.00)	0.61 (0.32-1.17)
**Face mask type used**				
Cloth	Ref.	Ref.	Ref.	Ref.
Surgical	1.18 (1.01-1.39)	1.14 (0.96-1.35)	1.47 (1.18-1.82)	1.95 (1.25-3.05)
FFP2	1.21 (1.00-1.45)	1.34 (1.11-1.62)	2.05 (1.62-2.60)	2.31 (1.46-3.64)
**Face mask use in family meetings**				
No	Ref.	Ref.	Ref.	Ref.
Sometimes	1.00 (0.83-1.21)	1.15 (0.93-1.43)	1.08 (0.82-1.43)	1.70 (1.25-2.31)
Yes	1.14 (1.01-1.28)	1.25 (1.09-1.43)	1.20 (0.99-1.46)	1.25 (0.97-1.62)
Did not meet	1.09 (0.94-1.26)	1.52 (1.31-1.77)	1.30 (1.03-1.63)	1.24 (0.92-1.67)
**Indoor bar attendance**				
<Once/month	Ref.	Ref.	Ref.	Ref.
1-3 times/month	1.05 (0.90-1.22)	0.98 (0.83-1.16)	0.79 (0.64-0.97)	0.92 (0.71-1.21)
Once or more/week	1.09 (0.93-1.27)	0.87 (0.71-1.05)	0.82 (0.64-1.05)	0.92 (0.66-1.28)
**Indoor family meetings with >10 people**				
Never	Ref.	Ref.	Ref.	Ref.
1-5 times	0.97 (0.81-1.17)	0.87 (0.72-1.05)	0.77 (0.62-0.97)	0.93 (0.71-1.21)
>5 times	1.25 (0.97-1.60)	0.74 (0.51-1.05)	0.73 (0.47-1.14)	0.94 (0.51-1.76)
**Indoor events, most people without mask**				
No	Ref.	Ref.	Ref.	Ref.
Yes	0.92 (0.75-1.14)	0.92 (0.76-1.11)	0.78 (0.61-1.01)	0.79 (0.57-1.09)

[a]The 2020 campaign was not finished when the survey took place; in this table, participants answering that they had still not received the vaccine but intended to be vaccinated were considered vaccinated. [b]Risk conditions: diabetes; lung, liver or kidney chronic disease; asthma, cardiovascular disease, hematological neoplasm, any other cancer in the last five years, immunosupressor treatment, or severe obesity. SPR: Standardized prevalence ratio (standardized to the overall distribution of sex, education, and Autonomous Community).

Table S3. Influenza vaccination coverage in 2019 and 2020[a] and coverage differences among adults of selected vaccination target groups[b], excluding participants that intended but had still not received the 2020 vaccine (ENE-COVID study, Spain, November 2020).

	N	2019 coverage, % (95% CI)[c]	2020 coverage, % (95% CI)[c]	Standardized difference (95% CI)[c]
Total	27,011	31.2 (30.3-32.1)	42.2 (41.2-43.3)	11.0 (9.6-12.4)
Influenza-related characteristics				
**Influenza vaccination target group**				
≥65 yeats	9740	59.1 (57.5-60.6)	73.1 (71.7-74.5)	14.0 (11.9-16.1)
<65 with a risk condition[d]	7270	24.3 (22.9-25.6)	34.5 (32.9-36.1)	10.3 (8.2-12.3)
Health care workers	2000	22.3 (19.5-25.2)	40.2 (36.9-43.5)	17.9 (13.6-22.3)
Living with someone with a risk condition[d]	7678	8.2 (7.3-9.0)	14.9 (13.7-16.1)	6.7 (5.3-8.2)
Security and emergency workers	323	8.4 (4.4-12.4)	14.3 (9.3-19.3)	5.9 (-0.5-12.3)
**Number of influenza vaccination criteria**				
1	15,410	27.4 (26.1-28.6)	39.4 (38.1-40.8)	12.0 (10.2-13.9)
2	8680	32.8 (31.4-34.1)	43.8 (42.3-45.4)	11.1 (9.0-13.2)
3	2921	40.8 (38.2-43.4)	51.9 (48.8-55.1)	11.2 (7.1-15.3)
Sociodemographic characteristics				
**Sex**				
Men	12,340	30.8 (29.8-31.9)	40.9 (39.6-42.1)	10.0 (8.4-11.7)
Women	14,671	31.5 (30.4-32.6)	43.4 (42.2-44.6)	11.9 (10.3-13.5)
**Age group**				
18-34	3828	9.5 (8.2-10.8)	14.8 (13.1-16.4)	5.3 (3.2-7.4)
35-59	10,927	15.9 (14.9-16.9)	25.8 (24.5-27.0)	9.9 (8.2-11.5)
60-64	2516	35.2 (32.5-37.8)	52.6 (49.8-55.5)	17.5 (13.6-21.3)
65-69	3135	47.7 (45.1-50.2)	68.6 (66.4-70.8)	20.9 (17.5-24.3)
70-74	2651	56.5 (53.8-59.3)	72.7 (70.1-75.3)	16.2 (12.4-20.0)
75-79	1893	65.7 (62.7-68.6)	74.2 (71.5-77.0)	8.5 (4.5-12.5)
≥80	2061	74.6 (71.8-77.5)	79.9 (77.4-82.4)	5.3 (1.5-9.0)
**Nationality**				
Spanish	26,255	31.4 (30.5-32.3)	42.6 (41.5-43.7)	11.2 (9.8-12.6)
Other	756	23.1 (19.0-27.3)	29.2 (25.1-33.4)	6.1 (0.3-12.0)
**Education**				
Primary or less	7402	35.1 (33.6-36.6)	45.0 (43.2-46.7)	9.9 (7.6-12.2)
High school/vocational education	14,129	28.7 (27.6-29.8)	39.9 (38.5-41.2)	11.2 (9.4-12.9)
University	5284	31.7 (29.8-33.6)	44.9 (43.0-46.8)	13.2 (10.5-15.9)
**Employment status**				
Actively working	10,831	22.9 (21.2-24.5)	34.5 (32.7-36.3)	11.7 (9.2-14.2)
Student	920	18.3 (13.4-23.2)	23.6 (18.5-28.8)	5.3 (-1.8-12.5)
Retired	10,049	39.1 (37.4-40.9)	53.2 (51.1-55.3)	14.1 (11.3-16.8)
Unemployed/other	5211	29.2 (27.4-30.9)	39.9 (38.0-41.8)	10.7 (8.1-13.3)
**On-site activity**				
No	15,256	34.8 (33.6-36.0)	46.6 (45.2-48.0)	11.8 (10.0-13.7)
On-site work	10,007	24.9 (23.3-26.6)	37.3 (35.6-39.0)	12.4 (10.0-14.8)
Other on-site activity	1748	24.2 (20.9-27.5)	31.1 (27.7-34.4)	6.9 (2.2-11.5)
Household and residence characteristics				
**Living with older adults**				
No	17,393	29.2 (28.2-30.3)	39.7 (38.5-40.8)	10.4 (8.9-12.0)
Yes	9604	34.4 (33.0-35.8)	47.3 (45.6-49.0)	12.9 (10.7-15.1)
**Living with children <3 years**				
No	25,918	31.2 (30.2-32.1)	42.3 (41.2-43.4)	11.1 (9.7-12.6)
Yes	1093	32.4 (28.2-36.6)	40.8 (36.7-45.0)	8.4 (2.5-14.3)
**Household size**				
1-2 people	11,060	32.2 (31.0-33.4)	44.0 (42.5-45.4)	11.8 (9.9-13.7)
≥3 people	15,951	30.3 (29.1-31.5)	41.0 (39.6-42.3)	10.7 (8.9-12.5)
**Municipality size (population)**				
>100,000	8034	31.4 (29.9-32.9)	42.6 (40.9-44.4)	11.2 (8.9-13.5)
20,000-100,000	7865	30.5 (29.0-32.0)	41.3 (39.5-43.1)	10.8 (8.5-13.1)
5000-20,000	5684	30.3 (28.6-32.1)	41.7 (40.0-43.4)	11.4 (8.9-13.8)
<5000	5428	33.1 (31.4-34.8)	43.8 (41.4-46.1)	10.7 (7.8-13.5)
**Relative income of the residential area**				
Under p25	7202	30.6 (29.1-32.1)	41.2 (39.3-43.1)	10.6 (8.1-13.0)
P25-50	6797	32.7 (31.0-34.4)	42.9 (41.0-44.8)	10.2 (7.6-12.7)
P50-75	6108	29.8 (28.3-31.2)	40.3 (38.4-42.2)	10.5 (8.1-12.9)
Over p75	6904	31.6 (29.7-33.5)	44.5 (42.5-46.6)	12.9 (10.2-15.7)
Health-related characteristics				
**Self-rated health**				
Very good or good	22,809	29.7 (28.8-30.7)	41.2 (40.1-42.3)	11.4 (10.0-12.9)
Bad or very bad	4202	38.3 (36.6-40.0)	48.0 (46.0-49.9)	9.7 (7.1-12.2)

	N	2019 coverage, % (95% CI)[c]	2020 coverage, % (95% CI)[c]	Standardized difference (95% CI)[c]
**Chronic diseases**				
None	10,055	22.2 (20.7-23.7)	33.8 (32.2-35.4)	11.6 (9.4-13.8)
1	7100	29.2 (27.9-30.6)	41.8 (40.2-43.3)	12.5 (10.4-14.6)
2-3	7840	37.5 (35.9-39.0)	49.1 (47.2-50.9)	11.6 (9.2-14.0)
≥4	2016	49.9 (47.0-52.8)	59.2 (56.2-62.2)	9.2 (5.1-13.4)
**Disability**				
No	24,900	30.5 (29.6-31.5)	41.6 (40.5-42.7)	11.0 (9.6-12.5)
Yes	1950	39.6 (36.8-42.3)	51.5 (48.5-54.5)	11.9 (7.9-16.0)
**Body mass index**				
Underweight and normal weight	10,235	29.4 (28.2-30.6)	40.2 (38.8-41.6)	10.8 (8.9-12.7)
Overweight	10,666	31.5 (30.3-32.8)	42.8 (41.4-44.2)	11.2 (9.4-13.1)
Obesity	6105	33.4 (31.9-35.0)	44.9 (43.1-46.6)	11.4 (9.1-13.8)
**Tobacco use**				
Never	13,897	31.7 (30.6-32.8)	42.3 (41.0-43.6)	10.6 (8.8-12.3)
Former	7502	34.3 (32.9-35.8)	47.9 (46.3-49.5)	13.6 (11.4-15.7)
Current	5612	24.6 (23.0-26.3)	34.4 (32.7-36.2)	9.8 (7.4-12.2)
COVID-19-related				
**Personal history**				
No infection	23,125	31.4 (30.4-32.4)	42.6 (41.5-43.7)	11.2 (9.7-12.7)
Not-severe infection	3209	29.6 (27.4-31.7)	39.6 (37.1-42.1)	10.0 (6.7-13.4)
Pneumonia or hospitalization	677	31.8 (28.0-35.6)	42.2 (37.5-46.9)	10.4 (4.3-16.4)
**Contact with case**				
No contact	17,406	31.8 (30.8-32.9)	42.2 (41.0-43.4)	10.4 (8.8-12.0)
Noncohabitant symptomatic contact	1168	27.1 (23.7-30.5)	39.9 (36.2-43.6)	12.8 (7.8-17.8)
Noncohabitant case contact	3988	30.4 (28.3-32.4)	43.4 (41.5-45.4)	13.0 (10.2-15.9)
Household symptomatic contact	1271	29.2 (25.6-32.8)	40.1 (36.5-43.7)	10.9 (5.9-15.9)
Household case contact	3178	30.8 (28.4-33.1)	42.5 (39.9-45.2)	11.8 (8.2-15.3)
**Cumulative provincial 7-day incidence (per 100,000)**				
<100	2152	27.0 (24.7-29.3)	37.1 (34.4-39.9)	10.1 (6.5-13.7)
100-200	17,067	31.8 (30.7-32.8)	42.5 (41.2-43.7)	10.7 (9.0-12.3)
200-300	7013	30.4 (28.6-32.2)	43.1 (41.0-45.2)	12.8 (10.0-15.5)
≥300	779	35.0 (31.2-38.8)	44.5 (40.2-48.8)	9.5 (3.7-15.2)
**Number of people in close contact**				
None	2865	33.7 (31.2-36.1)	43.8 (41.0-46.5)	10.1 (6.4-13.8)
1-2	8201	32.3 (30.9-33.6)	44.1 (42.4-45.7)	11.8 (9.7-13.9)
3-5	10,558	31.0 (29.7-32.3)	41.9 (40.4-43.3)	10.8 (8.9-12.8)
6-9	4006	28.7 (26.8-30.7)	41.1 (39.0-43.3)	12.4 (9.5-15.3)
≥10	1381	27.0 (23.6-30.4)	35.5 (32.2-38.8)	8.5 (3.7-13.2)
**Face mask type used**				
Cloth	4030	27.7 (25.9-29.6)	35.9 (33.9-37.9)	8.2 (5.5-10.9)
Surgical	17,254	30.5 (29.5-31.6)	42.1 (40.8-43.3)	11.5 (9.9-13.1)
FFP2	5673	35.1 (33.5-36.7)	47.0 (45.2-48.8)	11.9 (9.5-14.3)
**Face mask use in family meetings**				
No	9244	28.9 (27.6-30.2)	38.8 (37.4-40.2)	9.9 (8.0-11.8)
Sometimes	2409	29.5 (27.3-31.7)	40.7 (38.2-43.2)	11.2 (7.9-14.6)
Yes	11,048	33.1 (31.8-34.4)	44.9 (43.4-46.4)	11.8 (9.8-13.8)
Did not meet	4310	32.5 (30.7-34.3)	44.5 (42.4-46.5)	12.0 (9.3-14.7)
**Indoor bar attendance**				
<Once/month	20,166	32.5 (31.5-33.5)	43.3 (42.1-44.5)	10.8 (9.3-12.4)
1-3 times/month	3650	26.6 (24.7-28.6)	38.8 (36.7-40.9)	12.2 (9.3-15.0)
≥Once/week	3195	27.6 (25.4-29.8)	39.5 (37.1-42.0)	11.9 (8.6-15.3)
**Indoor family meetings with >10 people**				
Never	23,365	31.5 (30.6-32.5)	42.7 (41.6-43.8)	11.2 (9.7-12.6)
1-5 times	2885	28.3 (25.9-30.7)	38.8 (36.3-41.3)	10.5 (7.0-14.0)
>5 times	761	30.8 (26.5-35.1)	40.1 (35.8-44.5)	9.4 (3.2-15.5)
**Indoor events, most people without mask**				
No	24,673	31.4 (30.5-32.3)	42.6 (41.5-43.7)	11.2 (9.8-12.7)
Yes	2338	28.9 (26.4-31.5)	38.2 (35.5-40.9)	9.3 (5.5-13.0)
**Use of public transport**				
No	22,475	31.6 (30.7-32.6)	43.0 (41.9-44.2)	11.4 (9.9-12.9)
Yes	4536	29.5 (27.7-31.2)	39.1 (37.1-41.1)	9.6 (7.0-12.3)

[a]The 2020 campaign was not finished when the survey took place; in this table, participants answering that had they not still received the vaccine but intended to be vaccinated, were excluded. [b]Selected target groups: population older than 65 years, health care or sociosanitary workers or at home caregivers, population with some risk condition, living with someone with some risk condition, and security and emergency workers. [c]Standardized to the overall distribution of influenza vaccination group in the whole target population, except coverages for age groups that were not standardized. [d]Risk conditions: Diabetes, chronic lung, liver or kidney disease, asthma, cardiovascular disease, hematological neoplasm, any other cancer in the last five years, immunosupressor treatment or severe obesity.

Table S4. Prevalence of new influenza vaccination in 2020[a] among participants unvaccinated in 2019, by sociodemographic, health-related, and COVID-19-related factors, excluding those that intended but had still not received the 2020 vaccine (ENE-COVID study).

	N	Crude prevalence % (95% CI)	Standardized prevalence % (95% CI)[b]	Standardized prevalence difference % (95% CI)[b]	Standardized prevalence ratio (95% CI)[b]
Total	18,004	19.5 (18.6-20.5)			
Influenza related characteristics					
**Influenza vaccination target group**					
≥65 years	3923	39.2 (36.9-41.6)	39.5 (36.9-42.2)	Ref.	Ref.
<65 with risk a condition	5373	17.8 (16.3-19.3)	17.9 (16.5-19.4)	-21.6 (-24.5 to -18.7)	0.45 (0.41-0.50)
Health care workers	1507	26.5 (23.3-29.8)	24.6 (21.5-27.7)	-14.9 (-19.1 to -10.7)	0.62 (0.54-0.72)
Living with someone with a risk condition	6912	9.5 (8.5-10.5)	9.5 (8.5-10.6)	-30.0 (-32.9 to -27.1)	0.24 (0.21-0.27)
Security and emergency workers	289	10.6 (6.0-15.2)	11.7 (6.8-16.7)	-27.8 (-33.5 to -22.1)	0.30 (0.19-0.46)
**Number of influenza vaccination criteria**					
1	12,157	15.4 (14.5-16.4)	18.8 (17.7-19.9)	Ref.	Ref.
2	4841	26.9 (25.0-28.8)	20.3 (18.7-21.9)	1.5 (-0.4 to 3.4)	1.08 (0.98-1.19)
3	1006	37.6 (32.7-42.6)	22.4 (18.7-26.1)	3.6 (-0.3 to 7.5)	1.19 (1.00-1.42)
Sociodemographic characteristics					
**Sex**					
Men	8279	16.7 (15.6-17.8)	17.9 (16.8-19.1)	Ref.	Ref.
Women	9725	22.2 (21.0-23.4)	20.9 (19.8-22.0)	3.0 (1.7 to 4.3)	1.17 (1.09-1.25)
**Age**					
18-34	3404	9.1 (7.8-10.4)	8.9 (7.6-10.2)	Ref.	Ref.
35-59	9025	14.6 (13.5-15.7)	14.5 (13.4-15.5)	5.6 (4.0 to 7.1)	1.63 (1.40-1.90)
60-64	1652	31.1 (27.9-34.3)	31.6 (28.3-34.8)	22.7 (19.3 to 26.0)	3.55 (3.00-4.21)
65-69	1692	43.4 (40.0-46.8)	43.9 (40.5-47.3)	35.0 (31.3 to 38.7)	4.94 (4.18-5.84)
70-74	1106	41.9 (37.8-46.1)	42.9 (38.6-47.1)	34.0 (29.5 to 38.5)	4.82 (4.03-5.77)
75-79	629	31.2 (26.5-35.9)	32.6 (27.6-37.6)	23.7 (18.5 to 28.8)	3.67 (2.97-4.53)
≥80	496	29.0 (23.4-34.6)	30.8 (24.6-36.9)	21.9 (15.6 to 28.2)	3.46 (2.69-4.45)
**Nationality**					
Spanish	17,398	19.9 (19.0-20.9)	19.8 (18.8-20.7)	Ref.	Ref.
Other	606	10.0 (7.0-13.1)	12.8 (9.3-16.3)	-7.0 (-10.5 to -3.5)	0.65 (0.49-0.85)
**Education**					
Primary or less	3612	27.2 (25.1-29.4)	18.0 (16.4-19.6)	Ref.	Ref.
High school/vocational education	10,498	16.9 (15.8-18.0)	18.7 (17.5-19.9)	0.7 (-1.3 to 2.6)	1.04 (0.93-1.15)
University	3894	20.4 (18.6-22.2)	23.3 (21.4-25.2)	5.3 (2.7 to 7.9)	1.29 (1.14-1.47)
Household and residence characteristics					
**Living with older adults**					
No	12,714	16.6 (15.6-17.5)	18.1 (17.1-19.1)	Ref.	Ref.
Yes	5287	27.3 (25.4-29.1)	22.7 (21.1-24.4)	4.6 (2.8 to 6.5)	1.26 (1.15-1.37)
**Household size**					
1-2 people	6127	26.9 (25.2-28.6)	20.0 (18.6-21.4)	Ref.	Ref.
≥3 people	11,877	15.9 (14.9-17.0)	19.2 (18.1-20.4)	-0.7 (-2.5 to 1.0)	0.96 (0.88-1.05)
**Municipality size (population)**					
>100,000	5334	20.4 (18.9-21.9)	20.0 (18.6-21.5)	Ref.	Ref.
20,000-100,000	5386	18.4 (16.6-20.2)	19.2 (17.5-20.9)	-0.8 (-3.1 to 1.4)	0.96 (0.85-1.07)
5000-20,000	3894	19.0 (17.0-21.0)	19.1 (17.3-20.9)	-0.9 (-3.2 to 1.3)	0.95 (0.85-1.07)
<5000	3390	20.2 (17.9-22.6)	19.4 (16.8-21.9)	-0.7 (-3.6 to 2.3)	0.97 (0.83-1.12)
**Relative income of the residential area**					
Under p25	4844	18.5 (16.6-20.4)	19.8 (17.9-21.7)	Ref.	Ref.
P25-50	4465	18.8 (17.1-20.4)	19.3 (17.6-21.0)	-0.6 (-3.1 to 2.0)	0.97 (0.85-1.11)
P50-75	4146	18.5 (16.6-20.4)	18.3 (16.5-20.1)	-1.5 (-4.1 to 1.1)	0.92 (0.81-1.06)
Over p75	4549	22.6 (20.6-24.6)	20.8 (19.0-22.5)	0.9 (-1.6 to 3.4)	1.05 (0.92-1.18)
Health-related characteristics					
**Self-rated health**					
Very good or good	15,894	18.9 (18.0-19.9)	19.4 (18.4-20.4)	Ref.	Ref.
Bad or very bad	2110	24.4 (21.9-26.8)	20.4 (18.3-22.5)	1.0 (-1.2 to 3.2)	1.05 (0.94-1.17)
**Chronic diseases**					
None	8247	14.7 (13.6-15.8)	17.5 (16.1-18.9)	Ref.	Ref.
1	4771	22.6 (21.0-24.2)	20.6 (19.1-22.1)	3.1 (1.1 to 5.1)	1.18 (1.06-1.31)
2-3	4244	24.5 (22.7-26.3)	21.1 (19.3-22.9)	3.6 (1.2 to 6.0)	1.20 (1.06-1.36)
≥4	742	30.6 (26.2-34.9)	22.7 (19.1-26.3)	5.2 (1.3 to 9.2)	1.30 (1.08-1.56)
**Disability**					
No	16,957	19.2 (18.3-20.1)	19.4 (18.5-20.3)	Ref.	Ref.
Yes	1047	25.6 (22.0-29.2)	21.8 (18.5-25.0)	2.4 (-0.9 to 5.6)	1.12 (0.96-1.31)

	N	Crude prevalence<br>% (95% CI)	Standardized prevalence<br>% (95% CI)[b]	Standardized prevalence difference<br>% (95% CI)[b]	Standardized prevalence ratio<br>(95% CI)[b]
**Body mass index**					
Underweight and normal weight	7355	17.3 (16.0-18.5)	18.8 (17.5-20.1)	Ref.	Ref.
Overweight	6847	20.5 (19.1-21.9)	19.3 (18.0-20.6)	0.5 (-1.2 to 2.3)	1.03 (0.94-1.13)
Obesity	3799	22.8 (20.9-24.7)	21.3 (19.5-23.2)	2.5 (0.3 to 4.8)	1.13 (1.01-1.27)
**Tobacco use**					
Never	8965	19.8 (18.6-21.0)	19.6 (18.4-20.8)	Ref.	Ref.
Former	4522	26.2 (24.4-28.1)	22.3 (20.7-23.9)	2.7 (0.9 to 4.6)	1.14 (1.05-1.24)
Current	4517	12.9 (11.6-14.3)	16.2 (14.6-17.8)	-3.3 (-5.2 to -1.4)	0.83 (0.74-0.93)
COVID-19-related					
**Personal history**					
No infection	15,369	19.8 (18.8-20.8)	19.9 (18.9-20.9)	Ref.	Ref.
Not-severe infection	2211	17.4 (14.9-19.8)	17.3 (14.9-19.6)	-2.6 (-5.1 to -0.1)	0.87 (0.75-1.00)
Pneumonia or hospitalization	424	21.6 (16.1-27.0)	19.4 (14.6-24.2)	-0.5 (-5.2 to 4.3)	0.98 (0.76-1.25)
**Contact with case**					
No contact	10,908	20.2 (19.0-21.3)	18.4 (17.4-19.5)	Ref.	Ref.
Noncohabitant symptomatic contact	905	17.0 (13.4-20.5)	19.4 (15.4-23.4)	1.0 (-3.1 to 5.1)	1.05 (0.85-1.30)
Noncohabitant case contact	2986	19.7 (17.7-21.8)	22.3 (20.1-24.4)	3.8 (1.5 to 6.1)	1.21 (1.08-1.34)
Household symptomatic contact	958	15.9 (12.9-18.9)	19.7 (16.4-22.9)	1.2 (-2.2 to 4.6)	1.07 (0.90-1.27)
Household case contact	2247	19.1 (16.5-21.8)	21.5 (18.8-24.2)	3.1 (0.2 to 5.9)	1.17 (1.02-1.33)
**Cumulative provincial 7-day incidence (per 100,000)**					
<100	1532	16.8 (14.1-19.4)	31.1 (25.0-37.1)	Ref.	Ref.
100-200	11,367	19.2 (18.1-20.4)	18.9 (17.7-20.0)	-12.2 (-18.5 to -6.0)	0.61 (0.49-0.75)
200-300	4633	21.7 (19.8-23.7)	19.2 (17.0-21.4)	-11.9 (-18.5 to -5.3)	0.62 (0.49-0.78)
≥300	472	19.4 (14.6-24.3)	18.6 (13.8-23.4)	-12.5 (-19.7 to -5.3)	0.60 (0.44-0.81)
**Face mask type used**					
Cloth	3018	12.7 (11.2-14.3)	15.2 (13.5-17.0)	Ref.	Ref.
Surgical	11,391	20.4 (19.2-21.5)	19.8 (18.7-21.0)	4.6 (2.6 to 6.6)	1.30 (1.15-1.47)
FFP2	3576	23.2 (21.3-25.2)	21.9 (20.1-23.7)	6.6 (4.2 to 9.1)	1.44 (1.25-1.64)
**Face mask use in family meetings**					
No	6487	16.5 (15.2-17.8)	17.4 (16.2-18.7)	Ref.	Ref.
Sometimes	1733	18.2 (15.8-20.7)	20.1 (17.5-22.8)	2.7 (-0.3 to 5.6)	1.15 (0.99-1.34)
Yes	7092	21.7 (20.1-23.2)	21.1 (19.6-22.7)	3.7 (1.7 to 5.6)	1.21 (1.09-1.34)
Did not meet	2692	23.5 (21.2-25.8)	20.3 (18.4-22.2)	2.8 (0.6 to 5.1)	1.16 (1.04-1.31)
**Indoor bar attendance**					
<Once/month	12,829	20.6 (19.6-21.7)	19.6 (18.6-20.7)	Ref.	Ref.
1-3 times/month	2784	17.0 (15.0-19.0)	19.1 (17.1-21.2)	-0.5 (-2.8 to 1.8)	0.97 (0.87-1.10)
≥Once/week	2391	16.9 (14.7-19.1)	19.4 (17.1-21.7)	-0.2 (-2.7 to 2.2)	0.99 (0.87-1.12)
**Indoor family meetings with >10 people**					
Never	15,318	20.4 (19.4-21.4)	19.7 (18.8-20.7)	Ref.	Ref.
1-5 times	2147	14.9 (12.9-16.9)	18.1 (15.8-20.3)	-1.7 (-4.0 to 0.7)	0.92 (0.80-1.04)
>5 times	539	15.9 (11.4-20.3)	19.1 (14.6-23.6)	-0.6 (-5.2 to 3.9)	0.97 (0.76-1.23)
**Indoor events, most people without mask**					
No	16,237	20.2 (19.2-21.2)	19.7 (18.7-20.7)	Ref.	Ref.
Yes	1767	13.9 (11.7-16.2)	17.9 (15.2-20.6)	-1.8 (-4.6 to 1.0)	0.91 (0.78-1.06)

[a]The 2020 campaign was not finished when the survey took place; in this table, participants answering that they had still not received the vaccine but intended to be vaccinated were excluded. [b]Standardized to the overall distribution of influenza vaccination groups, sex, age, education, and Autonomous Community in the unvaccinated target population in 2019, except for influenza vaccination groups and age groups that were not mutually standardized.
